# IscR-mediated morphological regulation confers virulence and stress resistance by reducing stress molecule uptake in *Acinetobacter baumannii*

**DOI:** 10.1128/mbio.01871-26

**Published:** 2026-10-06

**Authors:** Hoan Van Ngo, Nayoung Kim, Jumi Pak, Jinki Yeom

**Affiliations:** 1 Department of Biomedical Science, College of Medicine, Seoul National University, Seoul, South Korea; 2 Department of Microbiology and Immunology, College of Medicine, Seoul National University, Seoul, South Korea; 3 Medical Research Center, Institute of Endemic Diseases, Seoul National University, Seoul, Republic of Korea; 4 Cancer Research Institute, Seoul National University 65492, Seoul, South Korea; South China Sea Institute of Oceanology, Guangzhou, Guangdong, China

**Keywords:** bacterial morphology, oxidative stress, antibiotics, *Acinetobacter baumannii*, peptidoglycan, surface area, bacterial stress response

## Abstract

Living organisms must adequately respond to stress to survive and proliferate. Bacterial pathogens face multiple stressors during infections, including oxidative stress from host innate immune cells and antibiotic treatment from clinical therapy. The pathogenic bacterium *Acinetobacter baumannii* is considered the most critical threat to public health due to its broad antibiotic resistance. However, it is poorly known how *A. baumannii* properly responds to antibiotics and stress molecules during infection. Here, we investigate the mechanisms by which *A. baumannii* regulates its morphology to reduce the uptake of stress molecules under oxidative stress and antibiotic exposure, thereby conferring virulence and survival during infection. The transcriptional regulator IscR responds to oxidative stress by upregulating *mrcA*, which encodes a PBP1a enzyme involved in peptidoglycan biosynthesis. Under oxidative stress, bacteria undergo a morphological shift from a rod to a coccoid form, reducing their surface area and thus decreasing their absorption of reactive oxygen species. Inactivation of either *iscR* or *mrcA* results in an elongated morphology characterized by an elevated surface area, thereby reducing *A. baumannii* survival under oxidative stress. Furthermore, IscR-mediated morphological control is essential for survival under antibiotic treatment. Moreover, IscR-mediated morphological regulation is required for *A. baumannii* survival in macrophage and mouse models. These findings elucidate a strategy by which *A. baumannii* uses IscR to adapt to stress through morphological control, facilitating its survival during infections against both immune response and antibiotic therapy.

**IMPORTANCE:**

*Acinetobacter baumannii* is a major cause of nosocomial infections. It poses a critical threat due to its extensive antibiotic resistance. This study reveals that the pathogen can change its cellular shape to survive immune system attacks and antibiotic treatment. This change represents a previously unknown survival strategy. *A. baumannii* transitions to a coccoid morphology under oxidative stress and antibiotic treatment. It does so by activating the *mrcA* gene, which encodes a peptidoglycan synthase enzyme, through the IscR transcriptional regulator. This rapid morphological adaptation helps *A. baumannii* evade host defenses and resist antibiotic treatment by reducing uptake of stress molecules. Our findings advance understanding of how pathogens adapt to hostile environments and identify new therapeutic targets. By blocking this shape-remodeling ability, it may be possible to render pathogenic bacteria more vulnerable to immune responses and antimicrobial treatments. This offers a promising strategy for combating this multidrug-resistant pathogen.

## INTRODUCTION

All living cells meticulously change their shape in response to environmental conditions (1–5). For instance, they detect ambient osmolarity and subsequently alter their cellular shape by regulating transcription, translation, metabolism, and membrane dynamics (1–5). Bacteria can alter their cellular shape to resist stressful environments by regulating elongation, division, and cell wall synthesis pathways (1–5). During infections, pathogenic bacteria encounter harsh conditions in the host, including oxidative stress induced by reactive oxygen species (ROS) produced by innate immune cells and antibiotic treatment during clinical therapy (6–11). Thus, regulation of cellular shape in response to ROS and antibiotics is essential for bacterial survival during infections (12–15). Nonetheless, it remains largely unexplored how pathogens modulate their morphology in response to various stress environments during infection. In this study, we show that pathogenic bacteria rapidly alter cellular morphology in response to stress conditions to enhance survival under antibiotic exposure and during host immune responses.

Bacteria can alter their morphology to endure stress and adapt to environmental changes (1–5). The bacterial cell wall, composed of peptidoglycan, is crucial for preserving cell shape and viability (16). In proteobacteria, the elongasome and divisome are two multiprotein complexes that coordinate peptidoglycan production and assembly, thereby regulating bacterial cell length and the development of daughter cells (17, 18). The class A penicillin-binding proteins (aPBPs), PBP1a and PBP1b, are bifunctional enzymes with both glycosyltransferase and D,D-transpeptidase (D,D-TPase) activities, which play a significant role in the synthesis of the cell wall and septal peptidoglycan (16). In *Escherichia coli*, PBP1a and PBP1b exhibit functional redundancy in regulating cell morphology, as the deletion of either protein does not affect bacterial morphology (19, 20). By contrast, in *Acinetobacter baumannii*, which poses a particular risk to immunocompromised patients and is associated with hospital outbreaks due to its high rate of antibiotic resistance (21–25), PBP1b is unable to substitute for the function of PBP1a (26). *A. baumannii* PBP1a collaborates with PBP3 to synthesize septal peptidoglycan, which is crucial for cell division and the formation of daughter cells (26). Although the biochemical functions of aPBPs in peptidoglycan synthesis in *A. baumannii* have been characterized, their physiological role in the bacterial stress response remains unclear.

In bacteria, the transcriptional regulator IscR is an essential component of the *isc* operon for the biogenesis of iron-sulfur (Fe-S) clusters (27, 28). IscR utilizes Fe-S clusters as cofactors to respond to oxidative stress (29–32). Under normal conditions, holo-IscR, which contains the Fe-S cluster, represses the *isc* operon (27, 28). The holo-IscR binds to both type I and type II DNA motifs in promoter regions of target genes (30). Oxidative stress causes damage to the Fe-S cluster in IscR, resulting in the formation of apo-IscR. Apo-IscR activates the *isc* operon and initiates Fe-S cluster biogenesis, restoring cellular homeostasis (27, 32, 33). Moreover, apo-IscR exclusively binds to type II DNA motifs in target promoter regions (28, 33–35). Apo-IscR directly activates the *sufA* operon, an alternative Fe-S cluster biogenesis system in *E. coli* (33), and the hemolysin synthase *vvhBA* operon in *Vibrio vulnificus* (36). Thus, IscR is a crucial regulator of stress responses and virulence in diverse bacterial pathogens (32, 35–38). Nevertheless, the pathogenic role of IscR in various pathogens, including *A. baumannii,* remains unexamined, despite the bacterium’s significance in antibiotic resistance and clinical settings.

This study demonstrates that *A. baumannii* IscR directly activates the expression of the *mrcA* gene, which encodes the PBP1a enzyme, in response to oxidative stress and antibiotic treatment. *A. baumannii* is a typically coccobacillary, rod-shaped bacterium, but IscR-mediated *mrcA* expression promotes bacterial morphology from rod shape to a coccoid morphology under oxidative stress and antibiotic treatment conditions. Coccoid morphology reduces the uptake of stress molecules, which enhances survival under oxidative stress and antibiotic exposure. Inactivation of *iscR* disrupts coccoid morphology, leading to the emergence of filamentous bacteria that are more vulnerable to oxidative stress and antibiotics. IscR-driven transition from rod to coccoid shape decreases surface area and stressor uptake under stress conditions. This morphological regulation via IscR is crucial for bacterial survival within macrophages and in murine infection models. Together, our findings reveal a novel regulatory mechanism in which pathogenic bacteria transcriptionally modulate their cellular morphology to reduce the uptake of stress molecules, thereby enhancing their survival and proliferation during host infections.

## RESULT

### IscR is required for defense against oxidative stress and virulence during infection by regulating cellular morphology

**Fig 1:**
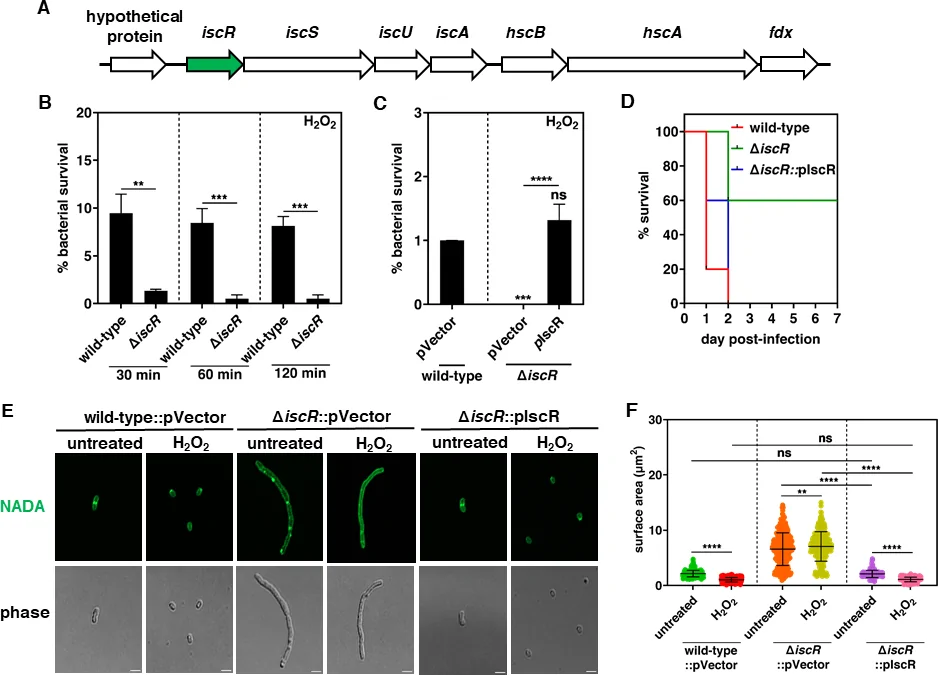
IscR-mediated morphological change in response to oxidative stress in *A. baumannii*. (**A**) Genetic map of the isc operon in *A. baumannii* where *iscR* (green color) is the first gene of the operon. (**B**) Percent survival of *A. baumannii* 17978 wild type and the *iscR* mutant (Δ*iscR*) after treatment with 10 mM H_2_O_2_ for 0, 30, 60, and 120 min. Data are presented as means ± SD from *n* = 3. *P* values were determined using an unpaired Student’s *t*-test. **P < 0.01 and ***P < 0.001. (**C**) Bacterial survival of *A. baumannii* 17978 wild type harboring the plasmid vector (pWH1266) and the *iscR* mutant (Δ*iscR*) harboring the plasmid vector or the *iscR*-expressing plasmid (pIscR) after treatment with 10 mM H_2_O_2_ for 2 h. Data are presented as means ± SD from *n* = 3. *P* values were compared between wild-type::pVector and other strains, or different combinations, using one-way ANOVA with Tukey’s multiple comparison test. ***P < 0.001; ****P < 0.0001; and ns, not significant. (**D**) Mouse survival curves infected with *A. baumannii* 17978 wild type harboring the plasmid vector and the *iscR* mutant (Δ*iscR*) harboring the plasmid vector or the *iscR*-expressing plasmid (pIscR). Six-week-old BALB/c mice (*n* = 6) were intraperitoneally challenged with 5 × 10^8^ colony-forming units/mL of bacteria, and survival time was monitored. (**E**) Fluorescence (upper) and phase (lower) microscopy of *A. baumannii* 17978 wild type harboring the plasmid vector (pVector) and the *iscR* mutant (Δ*iscR*) harboring the plasmid vector (pVector) or the *iscR*-expressing plasmid (pIscR) in normal conditions (untreated) or treated with 10 mM H_2_O_2_ for 30 min. Scale bar = 2 µm. (**F**) Surface area quantifications of each cell population (*n* ≥ 500) from panel **E** were calculated using ImageJ software with the MicrobeJ plugin. Each dot represents one cell. *P* values were determined using two-way ANOVA with Tukey’s multiple comparison test. **P < 0.01; ****P < 0.0001; and ns, not significant.

All domains of life require an iron-sulfur assembly system to generate iron-sulfur (Fe-S) clusters, which serve as critical cofactors for various enzymes (39–42). Reactive oxygen species can damage iron-sulfur clusters, leading to their disruption and subsequent loss of iron from enzymes. Bacteria have an Fe-S repair system, represented by the *isc* operon, to maintain the function of various enzymes (41, 43). *Acinetobacter baumannii*, a pathogenic bacterium, presents a high risk to immunocompromised patients due to its high rate of antibiotic resistance (44). The host immune system and antibiotic treatment cause oxidative stress (6–11). Given this, *A. baumannii* must overcome oxidative stress to cause disease in the host. Thus, we hypothesized that IscR, the master transcriptional regulator of the *isc* operon (Fig. 1A), is essential for the oxidative stress response and virulence during *A. baumannii* infections.

Multiple lines of evidence indicate that IscR is essential for oxidative stress defense and virulence in *A. baumannii*. First, the inactivation of *iscR* resulted in reduced bacterial survival under hydrogen peroxide (H_2_O_2_) exposure, known to be the cause of oxidative stress, in comparison to the wild type (Fig. 1B). Second, LIVE/DEAD staining showed a significant reduction in cell survival in H_2_O_2_-treated *iscR* mutant (~53%) compared to H_2_O_2_-treated wild type (~86%) (Fig. S1A and B). Bacteria treated with 70% ethanol were used as a positive control, as all bacteria were killed (Fig. S1A and B). Third, the survival defect of the *iscR* mutant in H_2_O_2_ was restored by a plasmid expressing *iscR* through a heterologous promoter. In contrast, the empty plasmid vector did not restore survival (Fig. 1C). Fourth, mice intraperitoneally inoculated with wild type showed a survival rate of 0% by day 2 (Fig. 1D, red line). By contrast, inactivation of *iscR* attenuated bacterial virulence, resulting in 60% survival in mice on day 7 post-infection (Fig. 1D, green line). The *iscR* mutant strain harboring a plasmid expressing *iscR* via a heterologous promoter fully restored the virulence of *A. baumannii* (Fig. 1D, blue line). Collectively, these findings indicate that IscR is required for survival under oxidative stress and virulence during infection in *A. baumannii*.

Then, we investigated how IscR contributes to oxidative stress defense and virulence in *A. baumannii*. In the presence of H_2_O_2_, rod-shaped cells transitioned to a coccoid morphology (Fig. 1E; Fig. S2A), as demonstrated by peptidoglycan staining with the fluorescent D-alanine derivative NADA [NBD-(linezolid-7-nitrobenz-2-oxa-1,3-diazol-4-yl)-amino-D-alanine] (NADA-green) that reduces bacterial surface area (Fig. 1F; Fig. S2B). NADA-green is integrated into the peptidoglycan of living bacteria during cell wall synthesis (45), indicating that the observed coccoid bacteria were viable. By contrast, the *iscR* mutant exhibited an elongated filamentous morphology with/without oxidative stress. Notably, because the *iscR* mutant has a single genome, as indicated by DAPI intensity similar to that in the wild type, with multiple septa or no fully formed septum (Fig. S3), inactivation of *iscR* impaired bacterial division, resulting in an elongated filamentous morphology. In addition, the *iscR* mutant failed to transition to a coccoid form under oxidative stress conditions (Fig. 1E; Fig. S2A) and reduce bacterial surface area (Fig. 1F; Fig. S2B). The defect in morphology transition ability was fully restored in the complemented *iscR* mutant (Fig. 1E and F), which enabled bacteria to survive better under oxidative stress conditions (Fig. 1C). Additionally, the filamentous morphology of the *iscR* mutant is more sensitive to oxidative stress than the coccoid form in wild type (Fig. 1B). Together, *A. baumannii* changes its morphology to coccoid to reduce its surface area via IscR, which enhances bacterial survival under oxidative stress and virulence during infection.

### IscR directly activates *mrcA* expression to regulate cellular morphology

Next, to investigate how IscR controls cellular morphology in *A. baumannii*, we focused on the class A penicillin-binding protein 1a (PBP1a), which is essential for division, cell shape, and cellular fitness in *A. baumannii* (26, 46). This requirement depends on the activity of PBP1a peptidoglycan synthase at the septum. However, it is unclear how *A. baumannii* controls the expression of the *mrcA* gene, which encodes the PBP1a enzyme, in response to stress. We found that the transcriptional regulator IscR is required for morphology regulation under oxidative stress (Fig. 1). Based on this, we reasoned that IscR controls *mrcA* expression during oxidative stress.

**Fig 2:**
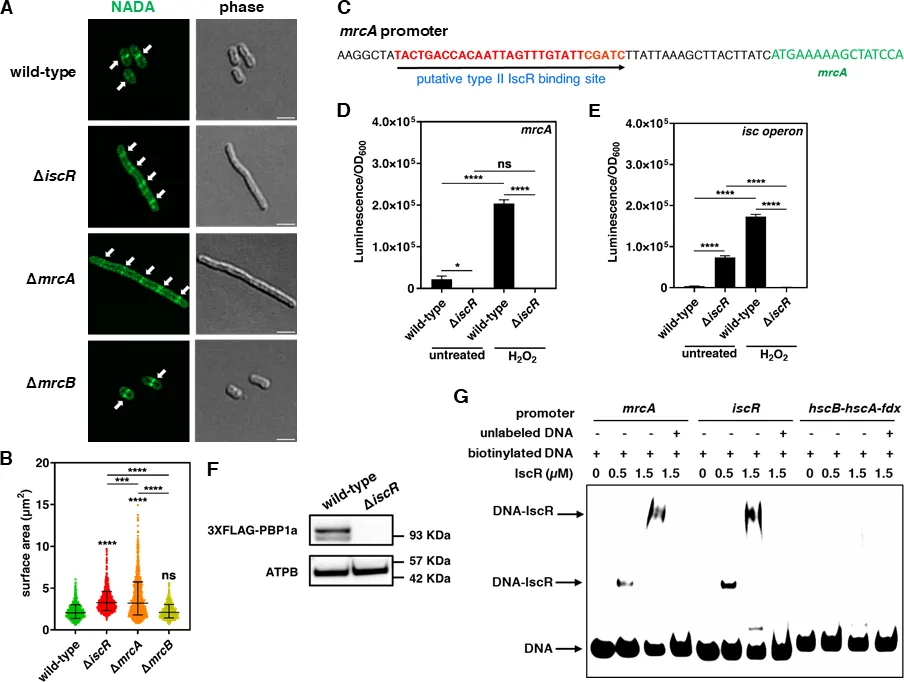
IscR regulates the expression of *mrcA* to maintain cell shape and respond to oxidative stress. (**A**) Fluorescence (left) and phase (right) microscopy of *A. baumannii* 17978 wild type, the *iscR* (Δ*iscR*), *mrcA* (Δ*mrcA*), and *mrcB* (Δ*mrcB*) mutants in normal conditions. Arrows indicate bacterial septa. Scale bar = 2 µm. (**B**) Surface area quantifications of each cell population (*n* ≥ 500) from panel **A** were calculated using ImageJ software with the MicrobeJ plugin. Each dot represents one cell. *P* values were compared between wild-type and other strains, or different combinations, using one-way ANOVA with Tukey’s multiple comparison test. ***P < 0.001; ****P < 0.0001; and ns, not significant. (**C**) Putative type II IscR binding site on the promoter region of *mrcA*. (**D**) Luminescence emission of *A. baumannii* 17978 wild type or the *iscR* mutant (Δ*iscR*) carrying the luciferase reporter plasmid pLPV1Z fused with the *mrcA* promoter at 2 h in the absence or presence of 10 mM H_2_O_2_. Data are presented as means ± SD from *n* = 3. *P* values were determined using two-way ANOVA with Tukey’s multiple comparison test. *P < 0.05; ****P < 0.0001; and ns, not significant. (**E**) Luminescence emission of *A. baumannii* 17978 wild type or the *iscR* mutant (Δ*iscR*) carrying the luciferase reporter plasmid pLPV1Z fused with the *isc* operon’s promoter at 2 h in the absence or presence of 10 mM H_2_O_2_. Data are presented as means ± SD from *n* = 3. *P* values were determined using two-way ANOVA with Tukey’s multiple comparison test. ****P < 0.0001; ns, not significant. (**F**) Western blot analysis of extracts prepared from *A. baumannii* 17978 harboring 3×FLAG-*mrcA* on the chromosome in wild-type strain and *iscR* mutant (Δ*iscR*). Samples were analyzed using an antibody directed to the FLAG epitope. ATPB was used as a loading control. Data are representative of three independent experiments, which gave similar results. (**G**) Electrophoretic mobility shift assay of DNA fragments carrying the *A. baumannii mrcA*, *isc* operon, and *hscB-hscA-fdx* operon promoter regions with purified IscR protein. Representative images from three independent experiments with similar results are shown.

Inactivation of either *iscR* or *mrcA* results in filamentous morphology (Fig. 2A) and increased cellular surface area (Fig. 2B), consistent with previous research on the role of PBP1a in the division of rod-shaped *A. baumannii* (26, 46). In contrast, the inactivation of *mrcB*, which encodes a different penicillin-binding protein PBP1b, did not affect the cellular morphology and surface area in *A. baumannii* (Fig. 2A and B). In addition, peptidoglycan staining with NADA-green dye revealed a multiseptated filamentous shape in *iscR* and *mrcA* mutants, unlike the wild-type strain and *mrcB* mutants (Fig. 2A) (26, 46). Notably, both the *iscR* and *mrcA* mutants have a single genome because they have multiple septa or no fully formed septa (Fig. S3A), and their DNA amounts are similar, as indicated by their DAPI intensities, which are similar to those of the wild type (Fig. S3B). By contrast, the wild type has single septa and a double genome during division (Fig. S3). This indicates that inactivation of *iscR* and *mrcA* impaired bacterial division, resulting in an elongated filamentous morphology.

Furthermore, a putative type II IscR binding site was detected upstream of the predicted transcriptional start site of *mrcA* (Fig. 2C), which resembles the *E. coli* IscR type II motif (33, 34, 47). Luciferase reporter assays indicated that *mrcA* expression was significantly increased under oxidative stress conditions (Fig. 2D). In addition, *mrcA* expression dramatically decreased in the *iscR* mutant compared to the wild-type strain in both normal and oxidative stress conditions (Fig. 2D). Thus, IscR-mediated regulation of *mrcA* expression contributes to rod-shaped maintenance in normal conditions and coccoid shape change to adapt and defend against oxidative stress. By contrast, the expression of the *isc* operon is significantly elevated in the *iscR* mutant strain under normal conditions but not under oxidative stress (Fig. 2E), consistent with previous findings (27). Moreover, the protein level of PBP1a, which is encoded by the *mrcA* gene, was greatly reduced in the *iscR* mutant compared to the wild type (Fig. 2F). Subsequently, the purified IscR protein binds to a DNA fragment harboring the promoter region of *mrcA* (Fig. 2G). Furthermore, excess unlabeled *mrcA* promoter DNA successfully competed out the labeled *mrcA* promoter DNA from binding (Fig. 2G). Given that the *A. baumannii isc* promoter has type I and II IscR binding sites (Fig. S4) like *Burkholderia* (48), purified IscR bound to the promoter region of the *isc* operon. In contrast, it did not bind to the promoter region of the *hscB-hscA-fdx* operon, which does not have a putative IscR binding site on the promoter region (Fig. 2G).

Finally, both *iscR* and *mrcA* mutants demonstrated increased minimal inhibitory concentrations (MICs) to all β-lactam antibiotics tested, except meropenem, compared with the wild-type strain and the *mrcB* mutant (Table 1). Most β-lactam antibiotics target the D,D-transpeptidase activity of PBP1a, except meropenem, which targets both D,D-TPase and L,D-TPase (46). Thus, the *iscR* mutant cannot produce the target protein, PBP1a (Fig. 2E), which inhibits bacterial survival to β-lactam antibiotics. In contrast, the MIC of the membrane-targeting antibiotic colistin remained unchanged (Table 1). Taken together, IscR directly binds the *mrcA* promoter and is required for *mrcA* expression, which is essential for regulating cellular morphology in response to oxidative stress.

**TABLE 1:** Antibiotic susceptibilities of *A. baumannii* 17978 and its mutants

Antimicrobial			MIC (mg/L) for strain			
Class	Name	Target	Wild type	Δ*iscR*	Δ*mrcA*	Δ*mrcB*
β-Lactams	Carbenicillin	D,D-TPase	16	32	32	16
	Cefoperazone	D,D-TPase	128	1,024	512	128
	Cefotaxime	D,D-TPase	32	64	64	32
	Mecillinam	D,D-TPase	64	256	128	64
	Meropenem	D,D-TPase and L,D-TPase	2	2	2	1
Lipopeptide	Colistin	Lipid A	2	2	2	2

### PBP1a-mediated coccoid transition reduces ROS uptake that enhances bacterial survival

Here, we determined that *A. baumannii* undergoes a morphological transition from rod to coccoid shape in response to oxidative stress via IscR-mediated PBP1a expression, thereby improving bacterial survival under these conditions (Fig. 1 and 2). Since ROS molecules, such as H_2_O_2_, can diffuse across bacterial membranes (49), we hypothesized that reduced bacterial surface area, as seen in a coccoid shape via PBP1a, may decrease uptake of oxidative stress molecules, thereby increasing tolerance to stress and bacterial survival.

**Fig 3:**
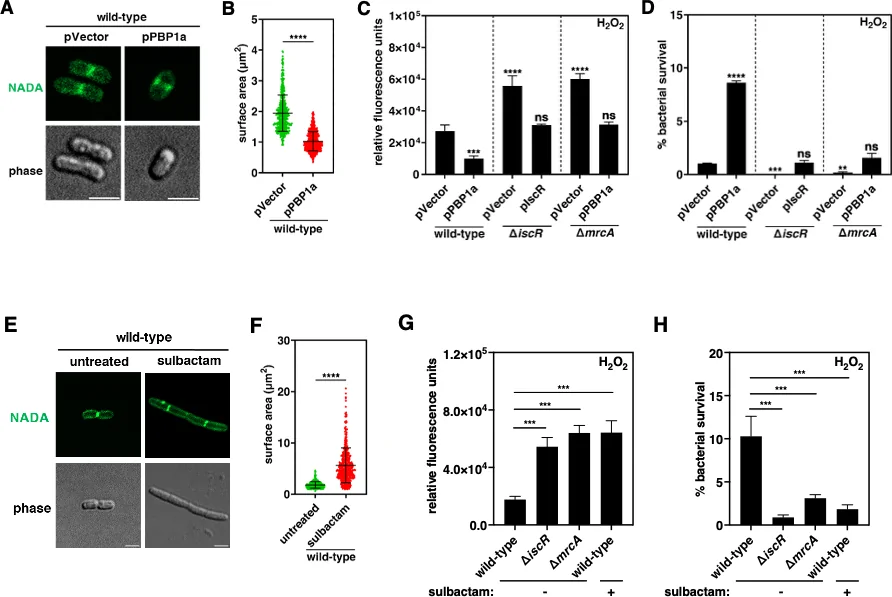
Sensitivity to oxidative stress depends on bacterial morphology. (**A**) Fluorescence (upper) and phase (lower) microscopy of *A. baumannii* 17978 wild type harboring the plasmid vector or the *mrcA*-expressing plasmid (pPBP1a) in normal conditions. Scale bar = 2 µm. (**B**) Surface area quantifications of each cell population (*n* ≥ 500) were calculated using ImageJ software with the MicrobeJ plugin. Each dot represents one cell. *P* values were determined using an unpaired Student’s *t*-test. ****P < 0.0001. (**C**) ROS uptake of *A. baumannii* 17978 wild type harboring the plasmid vector or the *mrcA*-expressing plasmid (pPBP1a), the *iscR* mutant (Δ*iscR*) harboring the plasmid vector or the *iscR*-expressing plasmid (pIscR), and the *mrcA* mutant (Δ*mrcA*) harboring the plasmid vector or the *mrcA*-expressing plasmid (pPBP1a) as measured by fluorescence intensity of DCF-DA upon treatment with 10 mM H_2_O_2_ for 30 min. The results are expressed as relative fluorescence units (RFUs) after subtracting the RFU value of the untreated control. Data are presented as means ± SD from *n* = 3. *P* values were compared between wild-type (pVector) and other strains using one-way ANOVA with Tukey’s multiple comparison test. ***P < 0.001; ****P < 0.0001; and ns, not significant. (**D**) Percent survival of *A. baumannii* 17978 wild type harboring the plasmid vector or the *mrcA*-expressing plasmid (pPBP1a), the *iscR* mutant (Δ*iscR*) harboring the plasmid vector or the *iscR*-expressing plasmid (pIscR), and the *mrcA* mutant (Δ*mrcA*) harboring the plasmid vector or the *mrcA*-expressing plasmid (pPBP1a) after treatment with 10 mM H_2_O_2_ for 2 h. Data are presented as means ± SD from *n* = 3. *P* values were compared between wild-type (pVector) and other strains using one-way ANOVA with Tukey’s multiple comparison test. **P < 0.01; ***P < 0.001; ****P < 0.0001; and ns, not significant. (**E**) Fluorescence (upper) and phase (lower) microscopy of *A. baumannii* 17978 wild type after treatment with a sublethal dose of sulbactam (0.5× MIC) for 2 h. Scale bar = 2 µm. (**F**) Surface area quantifications of each cell population (*n* ≥ 500) from panel **E** were calculated using ImageJ software with the MicrobeJ plugin. Each dot represents one cell. *P* values were determined using an unpaired Student’s *t*-test. ****P < 0.0001. (**G**) ROS uptake of *A. baumannii* 17978 wild type untreated or treated with a sublethal dose of sulbactam (0.5× MIC) for 2 h and the *iscR* (Δ*iscR*) and *mrcA* (Δ*mrcA*) mutants after treatment with 10 mM H_2_O_2_ for 30 min. Data are presented as means ± SD from *n* = 3. *P* values were compared between wild-type and other strains, or different combinations, using one-way ANOVA with Tukey’s multiple comparison test. ***P < 0.001. (**H**) Percent survival of *A. baumannii* 17978 wild type untreated or treated with a sublethal dose of sulbactam and the *iscR* (Δ*iscR*) and *mrcA* (Δ*mrcA*) mutants after treatment with 10 mM H_2_O_2_ for 2 h. Data are presented as means ± SD from *n* = 3. *P* values were compared between wild-type and other strains, or different combinations, using one-way ANOVA with Tukey’s multiple comparison test. ***P < 0.001.

To investigate this hypothesis, we constructed bacterial strains harboring a plasmid expressing *mrcA* via a heterologous promoter. The coccoid cell shape and reduced cellular surface were observed in strains harboring a plasmid expressing *mrcA* under a heterologous promoter, in the absence of stress (Fig. 3A and B), similar to the coccoid morphology induced by ROS (Fig. 1E and F; Fig. S2). Notably, the coccoid shape of bacteria significantly reduces the uptake of ROS, as indicated by measurements using intracellular ROS indicator, 2′7′-dichlorodihydrofluorescein diacetate (DCFH-DA) (Fig. 3C), thereby enhancing bacterial survival (Fig. 3D). Subsequently, we treated with a sublethal dose of sulbactam, which is a class A β-lactamase inhibitor that targets both PBP1 and PBP3 in *A. baumannii* (50), to induce filamentous shape (Fig. 3E and F) like *iscR* or *mrcA* mutant strain (Fig. 2A). These cells exhibited significantly increased ROS uptake (Fig. 3G). They demonstrated dramatically increased susceptibility to oxidative stress (Fig. 3H). Of note, a sublethal dose of sulbactam did not significantly produce ROS compared with the untreated condition (Fig. S5). Thus, sulbactam-treated bacteria are more sensitive to H_2_O_2_ due to more uptake of exogenous H_2_O_2_, but not sulbactam-mediated ROS induction. Together, PBP1a-mediated morphological transition to a coccoid shape reduced the uptake of stress molecules that increase *A. baumannii* survival under oxidative stress.

### PBP1a is sufficient to rescue filamentous morphology and bacterial survival by inactivating *iscR*

IscR directly activates *mrcA* expression (Fig. 2), thereby maintaining coccoid shape under oxidative stress (Fig. 1). PBP1a-mediated coccoid cell shape decreases the uptake of ROS and enhances bacterial resistance to oxidative stress (Fig. 3). Thus, we examined whether PBP1a can restore filamentous to coccoid shape in the *iscR* mutant strain to enhance bacterial survival by decreasing cell surface area during oxidative stress.

**Fig 4:**
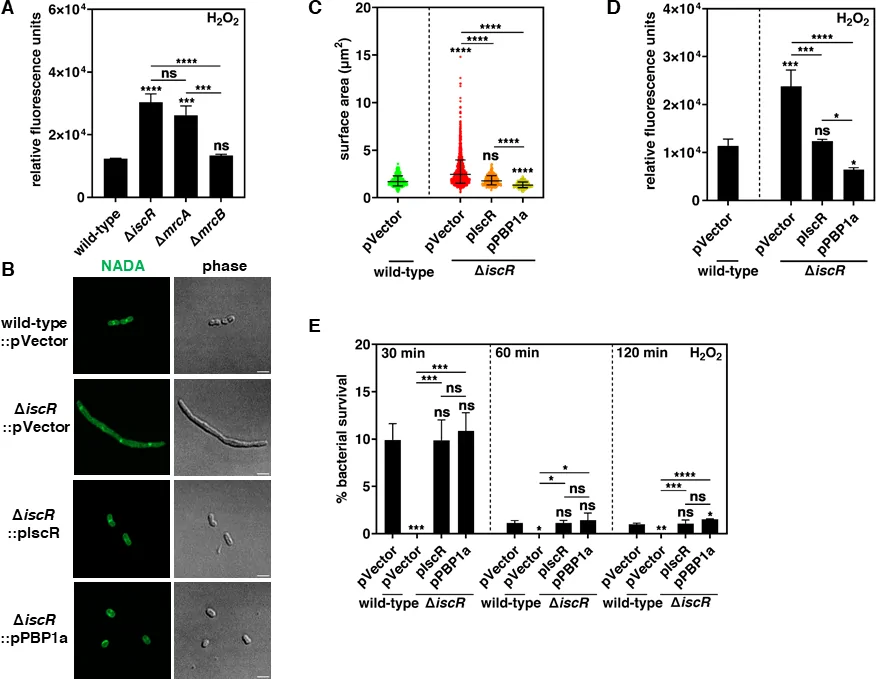
Complementation of the *iscR* mutant with *mrcA* rescues the defect in survival of the *iscR* mutant under oxidative stress by shortening its morphology. (**A**) ROS uptake of *A. baumannii* 17978 wild-type strain, the *iscR* (Δ*iscR*), *mrcA* (Δ*mrcA*), and *mrcB* (Δ*mrcB*) mutants upon treatment with 10 mM H_2_O_2_ for 30 min. The results are expressed as relative fluorescence units (RFUs) after subtracting the RFU value of the untreated control. Data are presented as means ± SD from *n* = 3. *P* values were compared between wild-type and other strains, or different combinations, using one-way ANOVA with Tukey’s multiple comparison test. ***P < 0.001; ****P < 0.0001; and ns, not significant. (**B**) Fluorescence (left) and phase (right) microscopy of *A. baumannii* 17978 wild type harboring the plasmid vector (pVector) and *iscR* harboring the plasmid vector (pVector), the *iscR*-expressing plasmid (pIscR), or the *pbb1a*-expressing plasmid (pPBP1a) in normal conditions. Scale bar = 2 µm. (**C**) Surface area quantifications of each cell population (*n* ≥ 500) from panel **B** were calculated using ImageJ software with the MicrobeJ plugin. Each dot represents one cell. *P* values were compared between wild-type::pVector and other strains, or different combinations, using one-way ANOVA with Tukey’s multiple comparison test. ****P < 0.0001; ns, not significant. (**D**) ROS uptake of *A. baumannii* 17978 wild type harboring the plasmid vector (pVector) and *iscR* mutant (Δ*iscR*) harboring the plasmid vector (pVector), the *iscR*-expressing plasmid (pIscR), or the *pbb1a*-expressing plasmid (pPBP1a) upon treatment with 10 mM H_2_O_2_ for 30 min. Data are presented as means ± SD from *n* = 3. *P* values were compared between wild-type (pVector) and other strains, or different combinations, using one-way ANOVA with Tukey’s multiple comparison test. *P < 0.05; ***P < 0.001; ****P < 0.0001; and ns, not significant. (**E**) Percent survival of *A. baumannii* 17978 wild type harboring the plasmid vector (pVector) and *iscR* mutant (Δ*iscR*) harboring the plasmid vector (pVector), the *iscR*-expressing plasmid (pIscR), or the *pbb1a*-expressing plasmid (pPBP1a) after treatment with 10 mM H_2_O_2_ for 0, 30, 60, and 120 min. Data are presented as means ± SD from *n* = 3. *P* values were compared between wild-type (pVector) and other strains, or different combinations, using one-way ANOVA with Tukey’s multiple comparison test. *P < 0.05; **P < 0.01; ***P < 0.001; ****P < 0.0001; and ns, not significant.

Filamentous morphology of *iscR* and *mrcA* mutants significantly increased in ROS uptake compared to the wild-type strain and *mrcB* mutant (Fig. 4A). The increased uptake of ROS is primarily due to exogenous H_2_O_2_ absorption, not changes in membrane permeability, which remained stable in the wild-type strain and *iscR* and *mrcA* mutants after H_2_O_2_ treatment (Fig. S6). The increased ROS uptake rendered the *iscR* and *mrcA* mutants more susceptible to oxidative stress (Fig. 1B; Fig. S7A). In contrast, inactivation of *mrcB* did not affect bacterial survival (Fig. S7A). In addition, the *mrcA* mutant with a plasmid expressing *mrcA* via a heterologous promoter restored bacterial survival under oxidative stress (Fig. S7B). In addition, the *iscR* mutant harboring a plasmid expressing *mrcA* via a heterologous promoter displayed a shortened cell shape (Fig. 4B) and reduced bacterial surface (Fig. 4C). As expected, the *iscR* mutant with a plasmid expressing *iscR* via a heterologous promoter demonstrated restored cellular morphology and cellular surface (Fig. 4B and C). Furthermore, the decrease in bacterial surface area due to the expression of *mrcA* and *iscR* genes in the *iscR* mutant strain led to reduced ROS uptake (Fig. 4D), which subsequently improved bacterial survival under oxidative stress (Fig. 4E). By contrast, the *iscR* mutant with a plasmid expressing *pbp3*, a different peptidoglycan synthase via a heterologous promoter, failed to correct the filamentous shape and bacterial survival observed in the *iscR* mutant (Fig. S8A and B). In summary, PBP1a is sufficient to restore the filamentous cell shape and increased bacterial surface area of the *iscR* mutant strain, thereby decreasing ROS uptake and enhancing bacterial survival.

### IscR-mediated *mrcA* expression reduces the surface area that enhances survival under antibiotic treatment

Bacteria can encounter various stresses, including oxidative stress and antibiotic exposure, during infections. These stress molecules must penetrate the bacterial membrane to inhibit bacterial survival and growth. Most antibiotics target cytoplasmic components by penetrating the bacterial membrane, except for β-lactam antibiotics. Thus, we hypothesized that *A. baumannii* may reduce antibiotic uptake by decreasing cellular surface area via IscR-mediated *mrcA* expression, as with ROS.

**Fig 5:**
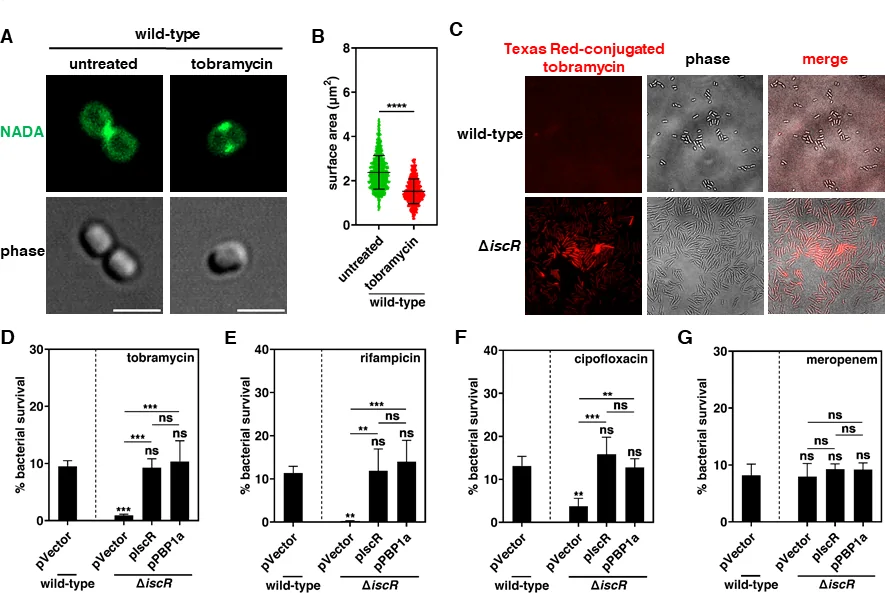
IscR-mediated morphology regulation is required for bacterial survival under antibiotic treatment. (**A**) Fluorescence (upper) and phase (lower) microscopy of *A. baumannii* 17978 wild type untreated or treated with 2× MIC of tobramycin for 30 min. Scale bar = 2 µm. (**B**) Surface area quantifications of each cell population (*n* ≥ 500) from panel **A** were calculated using ImageJ software with the MicrobeJ plugin. Each dot represents one cell. *P* values were determined using an unpaired Student’s *t*-test. ****P < 0.0001. (**C**) Uptake of Texas Red-conjugated tobramycin in *A. baumannii* 17978 wild type and the *iscR* mutant (Δ*iscR*) at 30 min post-incubation with 2× MIC of the conjugated antibiotic. Fluorescence microscopy images are representative from three independent experiments. (**D and E**) Percent survival of *A. baumannii* 17978 wild type harboring the plasmid vector (pVector) and the *iscR* mutant (Δ*iscR*) harboring the plasmid vector (pVector), the *iscR*-expressing plasmid (pIscR), or the *mrcA*-expressing plasmid (pPBP1a) after treatment with 2× MIC of tobramycin (**D**), rifampicin (**E**), ciprofloxacin (**F**), or meropenem (**G**) for 2 h. Data are presented as means ± SD from *n* = 4. *P* values were compared between wild-type (pVector) and other strains, or different combinations, using one-way ANOVA with Tukey’s multiple comparison test. **P < 0.01; ***P < 0.001; and ns, not significant.

Multiple lines of evidence support the notion that IscR-mediated *mrcA* expression reduces antibiotic uptake by decreasing cell surface area. First, *A. baumannii* transforms into a coccoid shape (Fig. 5A) and experiences a reduction in surface area (Fig. 5B) when exposed to tobramycin. This antibiotic binds to 30S and 50S ribosomal subunits in the cytoplasm (51). Second, the elongated shape of *iscR* inactivation allowed quick diffusion of tobramycin into the bacterial cytoplasm, compared to the rod-shaped wild-type strain (Fig. 5C). Third, the reduction in bacterial surface area resulting from PBP1a and IscR expression in the *iscR* mutant strain (Fig. 4B) enhanced bacterial survival against cytosolic antibiotics such as tobramycin, rifampicin, and ciprofloxacin (Fig. 5D through F). In contrast, the *iscR* mutant harboring a plasmid expressing *iscR* or *mrcA* under a heterologous promoter did not enhance survival upon treatment with meropenem, a β-lactam antibiotic (Fig. 5G). In conclusion, IscR-mediated expression of *mrcA* increases bacterial survival during antibiotic treatment by reducing the cell surface area and altering coccoid morphology. This indicates that regulation of cell morphology is critical for defense from various stress molecules, and this is not limited to ROS.

### IscR-mediated *mrcA* expression contributes to bacterial survival inside macrophages

Host innate immune cells, including macrophages and neutrophils, kill pathogenic bacteria through oxidative stress, including the production of nitric oxide and hydrogen peroxide (11). We demonstrated that IscR and PBP1a are required for oxidative stress defense by regulating cellular morphology (Fig. 1 and 4). Based on these findings, we reasoned that IscR and PBP1a are required for bacterial survival within host innate immune cells during infection.

**Fig 6:**
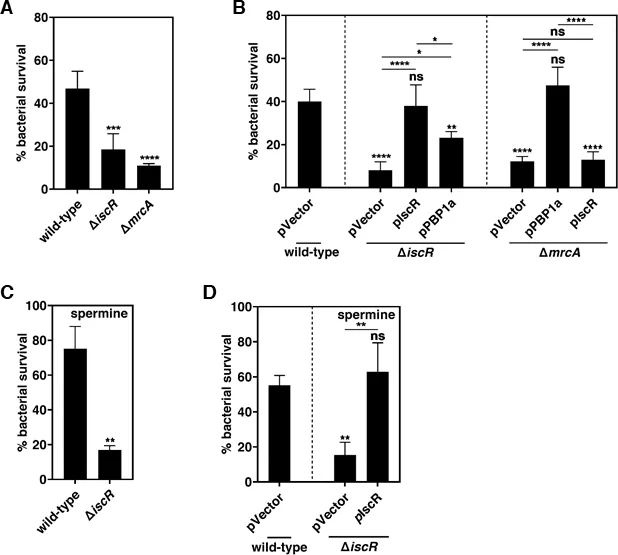
Both IscR and PBP1a are critical for bacterial survival against innate immune cells. (**A**) Bacterial survival of *A. baumannii* 17978 wild-type strain and the *iscR* (Δ*iscR*) and *mrcA* (Δ*mrcA*) mutants inside J774A.1 macrophages at 4 h post-infection. (**B**) Bacterial survival of *A. baumannii* 17978 wild type harboring the empty vector (pVector), the *iscR* mutant (Δ*iscR*) harboring the plasmid vector (pVector), the *iscR*-expressing plasmid (pIscR), or *mrcA*-expressing plasmid (pPBP1a), and the *mrcA* mutant (Δ*mrcA*) harboring the plasmid vector (pVector), the *mrcA*-expressing plasmid (pPBP1a), or *iscR*-expressing plasmid (pIscR) inside J774A.1 macrophages at 4 h post-infection. (**C**) Bacterial survival of *A. baumannii* 17978 wild-type strain and the *iscR* (Δ*iscR*) and *mrcA* (Δ*mrcA*) mutants after treatment with 1 mM spermine NONOate. (**D**) Bacterial survival of *A. baumannii* 17978 wild type harboring the plasmid vector (pVector), the *iscR* mutant (Δ*iscR*) harboring the plasmid vector or the *iscR*-expressing plasmid (pIscR), and the *mrcA* mutant (Δ*mrcA*) harboring the plasmid vector (pVector) or the *mrcA*-expressing plasmid (pPBP1a) after treatment with 1 mM spermine NONOate. Data are presented as means ± SD from *n* = 4 (**A and B**) or *n* = 3 (**C and D**). *P* values were compared between wild-type and other strains using one-way ANOVA with Dunnett’s multiple comparison test (**A**), or wild-type (pVector) and other strains and different combinations, using one-way ANOVA with Tukey’s multiple comparison test (**B and D**), or unpaired Student’s *t*-test (**C**). *P < 0.05; **P < 0.01; ***P < 0.001; ****P < 0.0001; and ns; not significant.

The survival rates of wild-type *A. baumannii* were approximately three- and fivefold higher than those of *iscR* and *mrcA* mutants within macrophages, respectively, at 4 h post-phagocytosis (Fig. 6A). In addition, bacterial survival was significantly enhanced in *iscR* or *mrcA* mutants harboring plasmids via expressing their respective genes from heterologous promoters (Fig. 6B). By contrast, isogenic strains with empty plasmid did not restore bacterial survival inside macrophages (Fig. 6B). Consistent with our findings (Fig. 4), expression of *mrcA* via heterologous promoters in the *iscR* mutant partially restored bacterial survival within macrophages (Fig. 6B). Because IscR is essential for bacterial survival by regulating genes necessary for adapting to multiple host-imposed stresses such as iron limitation and oxidative stress in *Vibrio vulnificus* (36) and *Salmonella enterica* (52), PBP1a expression partially rescues *A. baumannii* survival defect of the *iscR* mutant strain inside macrophages during infections. As expected, the expression of *iscR* via heterologous promoters in the *mrcA* mutant could not restore bacterial survival inside the macrophage (Fig. 6B), indicating that IscR directly regulated *mrcA* expression to survive during infections.

Additionally, the inactivation of *iscR* or *mrcA* reduced bacterial survival in spermine NONOate, a recognized generator of the nitrosative stress molecule nitric oxide during infections, compared to the wild type (Fig. 6C). The survival defect induced by spermine NONOate in the *iscR* or *mrcA* mutant strains was corrected by a plasmid expressing the *iscR* gene or *mrcA* gene, respectively (Fig. 6D). In contrast, the empty plasmid did not rescue bacterial survival (Fig. 6D). Altogether, IscR-mediated expression of *mrcA* is required for bacterial survival within host innate immune cells during infections.

## DISCUSSION

**Fig 7:**
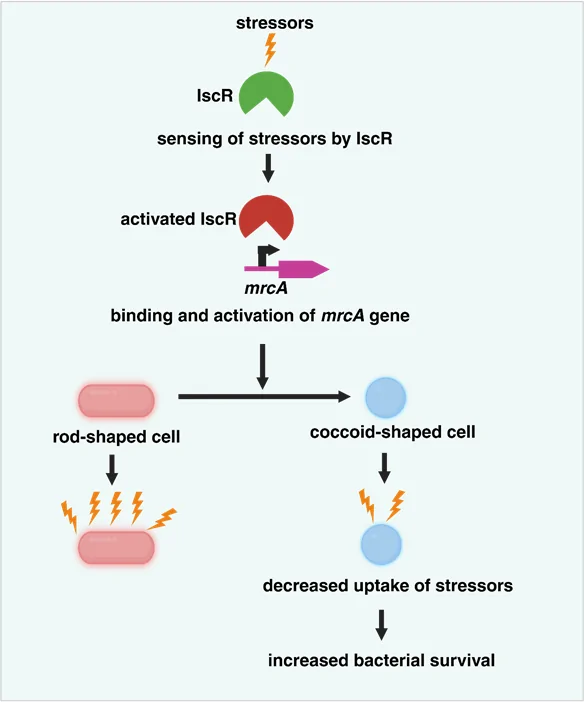
Regulation of PBP1a-mediated peptidoglycan synthesis by IscR in stress conditions. IscR activates *mrcA* expression in the presence of oxidative stress or antibiotics in *A. baumannii*. This promotes the transition from a rod shape to a coccoid shape in *A. baumannii*. The coccoid shape helps reduce uptake of stress molecules, thereby increasing bacterial survival within the host during infection and antibiotic treatment.

In this study, we demonstrate that the transcriptional regulator IscR protects *Acinetobacter baumannii* against oxidative stress (Fig. 1) and antibiotic treatment (Fig. 5) by regulating cellular morphology. *A. baumannii* is a rod-shaped coccobacillus (53), and IscR is crucial for maintaining its rod shape under normal conditions (Fig. 2). Surprisingly, IscR directly activates the expression of *mrcA*, a peptidoglycan synthase (Fig. 2) that facilitates the transition of cell shape from rod shape to coccoid shape in response to reactive oxygen species and antibiotics (Fig. 2–4). This morphological change reduces bacterial surface area, thereby restricting the uptake of stress molecules and increasing bacterial survival under stress conditions (Fig. 4 and 5). By contrast, inactivation of *iscR* or *mrcA* displayed filamentous morphology that enhanced stress-molecule uptake (Fig. 4). Notably, IscR-mediated morphology regulation is critical for pathogenesis inside macrophages (Fig. 6) and the murine model (Fig. 1D). Therefore, IscR-mediated remodeling of bacterial shape is critical for bacteria to enhance survival against immune defenses and antibiotic treatment during infection (Fig. 7).

IscR is a transcriptional regulator that controls the *isc* and *suf* operons required for the biogenesis of Fe-S clusters (30). Under normal conditions, IscR exists in both holo-IscR and apo-IscR, and the ratio between these forms is controlled by cellular Fe-S cluster demand (28, 31). Under oxidative stress conditions known to damage Fe-S clusters (54, 55), the holo-IscR levels decrease, which increases the level of apo-IscR (30). Even though both holo-IscR and apo-IscR can bind to the type II motif (31, 34, 47), promoters containing the type II motif are activated by apo-IscR under aerobic conditions (56). In this study, we identified the type II motif in the promoter region of the *mrcA* operon (Fig. 2C) and observed the binding of purified IscR to the *mrcA* promoter region (Fig. 2G). Since IscR was purified under aerobic conditions, most purified IscR would be apo-IscR (33, 57). Thus, apo-IscR may bind to the *mrcA* promoter type II motif region in *A. baumannii*. It would be interesting to investigate whether holo-IscR binds to the promoter region of the *mrcA* operon and regulates *mrcA* gene expression.

A previous study reported that PBP3 overexpression restores the septation defect of Δ*mrcA A. baumannii* (26). By contrast, expression of *pbp3* in the *iscR* mutant did not shorten the filamentous cells at all, nor did it improve survival under oxidative stress (Fig. S8). This difference may reflect the amounts of PBP3 produced, because we expressed *pbp3* from its native promoter, whereas the previous study used an inducible promoter (26). In addition, the two genetic backgrounds are not equivalent. The *mrcA* mutant lacks a single peptidoglycan synthase, whereas the *iscR* mutant lacks a transcriptional regulator that controls genes in addition to *mrcA*. In agreement with this notion, *mrcA* expression only partially restored survival of the *iscR* mutant inside macrophages (Fig. 6B). Notably, even in the Δ*mrcA* background, PBP3 overexpression rescued the division defect but not the fitness defect (26), indicating that restoration of septation is not sufficient to restore bacterial fitness. Although we cannot presently distinguish among these possibilities, our results are most consistent with IscR-mediated protection against oxidative stress requiring PBP1A specifically, rather than septal peptidoglycan synthesis by another synthase such as PBP3.

All three domains of life regulate cellular shape to adapt to environmental conditions. Although a decreased surface area, such as in a coccoid or round cell shape, generally reduces the efficiency of transporting materials between environments (58, 59), it also offers multiple advantages for organisms. In animals, a lower surface area is better at retaining heat, minimizing heat loss to the environment (60, 61). For instance, animals continuously exposed to cold temperatures decrease their surface area to reduce heat loss and maintain body temperature (60, 61). In addition, plants try to reduce their surface area to help prevent desiccation in dry periods (62, 63). Plants and insects reduce surface area to avoid water loss during osmotic stress (64, 65). Moreover, a reduced surface area can easily store more nutrients or energy than a large-surface-area cell shape under nutrient-limited conditions (3, 66), because a smaller surface area can protect its internal resources from external fluctuations. Therefore, surface reduction in unfavorable conditions is a widely conserved defense mechanism in all living organisms. Here, we investigated how bacteria reduce their surface area to inhibit the uptake of stress molecules, thereby protecting themselves from environmental stress (Fig. 7).

In many bacterial species, rod-shaped cells adopt a coccoid shape under stress conditions, including nutrient starvation, low pH, and the stationary phase (1–5). In *Pseudomonas aeruginosa*, coccoid cell formation induced by β-lactam antibiotics enables the bacterium to become more tolerant of carbapenem and penicillin antibiotics (67). Uropathogenic *Escherichia coli* also undergoes morphological changes from a rod to a coccoid shape inside the autophagosome of human bladder epithelial cells (68). Thus, the morphological transition from rod to coccoid shape is regarded as a bacterial survival strategy in response to stress. However, it is poorly understood how the coccoid shape contributes to the survival and pathogenesis of pathogenic bacteria during infection. This study demonstrates that viable coccoid cells reduce the uptake of stress molecules, such as ROS and antibiotics, by decreasing bacterial surface area through IscR-mediated PBP1a production in the pathogenic bacterium *Acinetobacter baumannii* (Fig. 2–4). Furthermore, this is required for survival and pathogenesis during infection in host innate immune cells (Fig. 6) and in the mouse model (Fig. 1D). Our findings suggest that the transition to a viable coccoid cell shape under unfavorable conditions may hinder the entry of stress molecules into the bacterial cytoplasm, a common strategy for the survival of pathogenic bacteria during infection (Fig. 7).

The emergence of multidrug-resistant bacteria poses a severe threat to public health, requiring the development of innovative strategies for antimicrobial therapy. Our findings indicate that drug combinations with conventional antibiotics and inhibitors for bacterial cell shape regulation may effectively eliminate bacteria. In a clinical setting, combination therapy with the β-lactamase inhibitor sulbactam and the β-lactam antibiotic ampicillin was widely used for decades to treat bacterial infections (69). Our study revealed that bacteria fail to transition to the coccoid form and remain filamentous under sulbactam treatment (Fig. 3E), and filamentous forms easily uptake antibiotics (Fig. 4). Thus, they are more valuable than coccoid cells under a β-lactam antibiotic treatment (Fig. 7). Consequently, the combination of sulbactam with other antibiotics is expected to demonstrate strong bactericidal effects against bacteria at concentrations where each individual drug shows minimal bactericidal activity. Notably, recent studies indicate that sulbactam combinations with various antibiotics, including colistin and durlobactam, enhance treatment outcomes for carbapenem-resistant *A. baumannii* (70–73), a pathogen the WHO has designated as the most critical (74). Therefore, our findings establish a framework for identifying novel antibacterial drug combinations to treat antibiotic-resistant bacterial infections by promoting the uptake of conventional antibiotics by pathogens.

## MATERIALS AND METHODS

### Bacterial strains and growth conditions

Bacterial strains and plasmids employed in this study are listed in Table S1. For routine cultivation and genetic manipulation, *A. baumannii* and *E. coli* were grown at 37°C in Luria-Bertani (LB) broth or on LB agar (BD Difco). For selection of recombinants or maintenance of plasmids in *A. baumannii* and *E. coli*, antibiotics were supplied at the following concentrations: ampicillin, 50 µg/mL; carbenicillin, 100 µg/mL; gentamicin, 10 µg/mL; and kanamycin, 50 µg/mL.

### Cell lines

The J774A.1 mouse macrophage cell line was cultured in Dulbecco’s Modified Eagle Medium High Glucose (Welgene) supplemented with 10% heat-inactivated fetal bovine serum (Welgene) at 37°C and 5% CO_2_.

### Mouse models

Wild-type C57BL/6 mice (Orient Bio, Seongnam, South Korea) were housed and maintained in the specific pathogen-free facility at Seoul National University College of Medicine.

### Construction of chromosomal mutants and plasmids in *A. baumannii*

Primers used in this study are listed in Table S2. Chromosomal mutants were generated using standard protocols described previously (75). Briefly, the FRT-flanked kanamycin resistance determinant of pKD4 was amplified using primers bearing ~50 bp of homology to the region flanking the gene of interest. This PCR product was electroporated into competent *A. baumannii* carrying the pAT02 plasmid, which expresses the RecAb recombinase. Mutants were selected on LB agar plates with kanamycin, and integration of the resistance marker was confirmed by PCR. To remove the kanamycin resistance cassette, electrocompetent mutants were transformed with the pAT03 plasmid, which expresses the FLP recombinase. All mutant strains were confirmed by PCR and sequencing.

Complementation of *iscR* or *mrcA* mutants was performed in *trans* using the vector pWH1266 (76). The gene and its native promoter were amplified using genomic DNA from wild-type ATCC 17978 as a template, and the primers are listed in Table S2. pWH1266 was digested with BamHI and SalI (New England Biolabs). The purified PCR product and cut plasmid were joined using NEB Builder HiFi Assembly (New England Biolabs) at 50°C for 15 min. The resulting assembly was transformed into chemically competent DH5α by heat shock, and successful transformants were selected on LB agar plates with ampicillin following overnight growth at 37°C. The constructs were confirmed by PCR and sequencing prior to the introduction of this vector, as well as the native empty vector, into the mutants. Successful transformants were selected on LB agar plates with ampicillin.

### Bacterial survival assays

Freshly streaked bacterial strains were grown overnight in LB broth at 37°C under shaking. Overnight cultures were subcultured at an OD_600_ of 0.005 in LB broth and grown to early-stationary phase (6 h). Bacteria were diluted to an OD_600_ of 0.5, washed twice with 1× phosphate-buffered saline (PBS; pH 7.4) (137 mM NaCl, 2.7 mM KCl, 10 mM Na_2_HPO_4_, and 1.8 mM KH_2_PO_4_). Then, bacteria were resuspended in PBS without or with drugs and incubated at 37°C with shaking for 2 h. The drugs include H_2_O_2_ and Spermine NONOate (GlpBio). The names of the drugs and their concentrations are indicated in the figures. The live bacterial numbers or colony-forming units (CFUs) were determined by serial dilution and plating. Percent bacterial survival was calculated by dividing the number of CFU/mL of drug-treated bacteria by the number of CFU/mL of untreated bacteria.

### Fluorescent NADA staining and image analysis

To visualize bacterial peptidoglycan, a previously described protocol has been used (26, 46, 77). Briefly, subcultured bacteria were washed once with LB broth, incubated with 3 µL of 10 mM NBD-(linezolid-7-nitrobenz-2-oxa-1,3-diazol-4-yl)-amino-D-alanine (Tocris Bioscience) at 37°C for 30 min, and fixed with 1× PBS containing 4% paraformaldehyde (Biosesang). DAPI was used for staining bacterial chromosomes. Fixed bacteria were immobilized on agarose pads and imaged using a Zeiss LSM 880 inverted confocal microscope or a Zeiss AxioObserver.Z1 inverted fluorescence microscope with a 63× oil immersion objective and a laser line of 488 nm for detection. All images were processed using ImageJ Fiji (78), and bacterial surface area was calculated as surface square pixels using MicrobeJ (79). DAPI intensity was also measured using MicrobeJ. At least 300 cells were analyzed for all experiments, and data were plotted using GraphPad Prism 10 (10.4.1). Each experiment was repeated three times, and each replication was used in the data set. One representative image from the data sets was included in each figure.

### Analysis of LIVE/DEAD staining by fluorescence microscopy

The viability of bacteria was determined using the LIVE/DEAD BacLight Bacterial Viability Kit (Thermo Fisher Scientific). Staining was performed according to the manufacturer’s instructions using the fluorescent dyes SYTO9 and propidium iodide (PI). Stained bacteria were immobilized on agarose pads and imaged using a Zeiss AxioObserver.Z1 inverted fluorescence microscope with a 63× oil immersion objective and laser lines of 488 and 555 nm for detection of SYTO9 and PI, respectively. All images were processed using ImageJ Fiji. At least 500 cells per data set were analyzed for all experiments. One representative image from the data sets was shown.

### Determination of minimum inhibitory concentration

The antibacterial activity of antibiotics against *A. baumannii* was evaluated using the broth microdilution method according to CLSI guidelines (80). Briefly, each well of a sterile 96-well microliter plate received 50 µL of LB broth containing increasing concentrations of a specific antibiotic. The bacterial inoculum was adjusted to an OD_600_ of 0.01, and 50 µL was added to each well. The plates were incubated at 37°C for 24 h, and bacterial growth was quantified by measuring OD_600_. The MIC was defined as the lowest concentration showing less than half the growth of untreated bacteria.

### Reporter assays

To construct reporter plasmids (pLPV1Z-P*_mrcA_* and pLPV1Z-P*_iscoperon_*), promoter regions of the *mrcA* operon and the *isc* operon*,* respectively, were amplified using the primers listed in Table S2 (81). pLPV1Z was digested with PstI and XbaI (New England Biolabs). The purified PCR product and cut plasmid were joined using NEB Builder HiFi Assembly (New England Biolabs) at 50°C for 15 min. The resulting assembly was transformed into chemically competent DH5α by heat shock, and successful transformants were selected on LB agar plates with gentamicin following overnight growth at 37°C. The constructs were confirmed by PCR and sequencing prior to introduction of this vector into wild-type strain and mutants. Successful transformants were selected on LB agar plates with gentamicin.

For experiments to check gene expression (Fig. 2D and E), bacterial strains harboring pLPV1Z-P*_mrcA_* or pLPV1Z-P*_isc operon_* were grown overnight in LB broth at 37°C under shaking. Overnight cultures were subcultured at an OD_600_ of 0.005 in LB broth and grown to early-stationary phase (6 h). A volume of 100 µL of bacterial culture was added to 100 µL of LB broth without and with 10 mM of H_2_O_2_ in 96-well white plates. The luminescence and OD_600_ values were recorded using the BioTek Synergy H1 plate reader.

### Western blot analysis

Protein concentrations were determined using the BCA Protein Assay Kit (Pierce), with bovine serum albumin used as a standard protein. Protein samples (100 µg of protein each) were run in a Bolt 4%–12% Bis-Tris Plus gel (Invitrogen) and transferred onto a nitrocellulose membrane using an iBlot 2 Gel Transfer Device (Invitrogen). Membranes were blocked with 5% skim milk solution at room temperature for 1 h. Then, samples were analyzed using anti-FLAG antibody (Sigma-Aldrich) (1:2,000 dilution) and secondary horseradish peroxidase-linked antibody (Cytiva) (1:5,000). The blots were developed with an ECL detection system (Thermo Scientific). Membranes were imaged using Amersham ImageQuant 900 (Cytiva).

### IscR protein purification

Full-length IscR of *A. baumannii* was obtained through PCR and inserted into the pET28b (+) vector with 6× His tag at the N-terminus. pET28B-IscR was transformed into BL21 (DE3) *E. coli* competent cells. The cells were grown overnight in LB broth at 37°C with shaking. The overnight culture was subcultured 1:100 in LB broth and grown at 37°C with shaking to an OD_600_ of 0.4. Culture was then induced with 1 mM isopropyl β-D-1-thiogalactopyranoside (Sigma-Aldrich) for 1 h at 30°C with shaking. Cells were harvested, and the pellet was resuspended in binding buffer (pH 7.4) containing Tris-buffered saline (TBS, Biosesang) and 10 mM imidazole (Sigma-Aldrich). Cells were lysed by sonication, and the resulting clarified supernatant was loaded onto an Econo-Pac Chromatography Column (Bio-Rad), which had been pre-loaded with Nickel Sepharose High Performance (Cytiva) and equilibrated with binding buffer. After overnight incubation at 4°C with rotation, the column was washed three times with wash buffer (pH 7.4) containing TBS and 40 mM imidazole. IscR protein was eluted using elution buffer (pH 7.4) containing TBS and 250 mM imidazole. Fractions containing pure protein were then collected after SDS-PAGE, combined, and concentrated using Amicon Ultra centrifugal filters (Merck Millipore). Protein concentration was determined using the BCA Protein Assay kit (Pierce) according to the manufacturer’s protocol.

### Electrophoretic mobility shift assay

Electrophoretic mobility shift assays (EMSA) were performed using the LightShift Chemiluminescent EMSA Kit (Thermo Scientific). Biotinylated DNA fragments from the upstream regions of *mrcA*, the *isc* operon, and the *hscB-hscA-fdx* operon were obtained by PCR using 5′-end biotin-labeled primers listed in Table S2. Binding reactions were incubated in 20 µL volume containing 25 fmol of biotinylated DNA and various concentrations of purified IscR protein (0–1.5 µM) in 1× Gel Shift Binding Buffer (Molecular Depot) for 60 min. To ensure binding specificity, a 50-fold molar excess of unlabeled competitor DNA was added to the reaction mixture where indicated. Binding reactions were loaded with the provided loading dye onto a 6% Novex TBE Gel (Invitrogen) and separated in 0.5× Novex TBE Running Buffer (Thermo Fisher) at 100 V for 60 min at 4°C. A Trans-blot Turbo Transfer System (Bio-Rad) was used to transfer proteins and DNA to Biodyne B positively charged nylon membranes (Thermo Fisher) for 30 min at 25 V. Cross-linking was performed by exposing the membranes to UV light for 15 min in a Dual LED Blue/White Light Transilluminator (Invitrogen). Blocking, incubation with streptavidin-horseradish peroxidase conjugate, washing, and revealing were performed according to the protocol. Membranes were imaged using Amersham ImageQuant 900 (Cytiva).

### ROS uptake assays

DCFH-DA was utilized as a fluorescent dye to detect ROS production upon treatment of bacteria with H_2_O_2_. Subcultured bacteria were washed with 1× PBS and treated with 10 µM DCFH-DA for 30 min at 37°C. Bacteria were then washed three times with 1× PBS to ensure the removal of excess fluorescent probes. Next, the probed bacteria were treated with 10 mM H_2_O_2_ for 30 min. After incubation, the fluorescence intensity was measured with excitation and emission wavelengths at 488 and 525 nm using the BioTek Synergy H1 plate reader.

### Outer membrane permeability assay

Outer membrane permeability was assessed using the fluorescent dye 1-N-phenyl-naphthylamine (NPN) (82). Bacteria were prepared and untreated or treated with H_2_O_2_ exactly in the same way as survival assays. After 2 h, bacteria were washed and resuspended in PBS before the addition of NPN solution (final concentration: 50 µM). After incubation for 30 min at 37°C in the dark, the fluorescence of NPN was measured with excitation and emission wavelengths at 350 and 420 nm, using the BioTek Synergy H1 plate reader.

### Texas Red-conjugated tobramycin uptake assays

To make Texas Red-conjugated tobramycin (TRT), Texas Red Sulfonyl Chloride (Invitrogen) was dissolved in anhydrous N, N-Dimethylformamide to 5 mg/mL on ice. Tobramycin (Sigma, 10 mg/mL) was mixed with Texas Red at a 1:1 volume ratio (100 µL each) in 0.1 M sodium bicarbonate buffer (800 µL, pH 8.5), followed by rotation and incubation on ice for 2 h in the dark. The reaction mixture was desalted to remove free dye, and the conjugate was diluted to 1.0 µg/mL for experimental use.

To investigate the time scale of antibiotic accumulation in living bacteria, subcultured bacteria were incubated with 1× MIC of TRT for 30 min, immobilized on agarose pads, and imaged using a Zeiss AxioObserver.Z1 inverted fluorescence microscope with a 63× oil immersion objective and a laser line of 555 nm for detection. All images were processed using ImageJ Fiji. One representative image from three independent experiments was shown.

### Intramacrophage survival assay

For the intracellular survival assays, J774A.1 cells were seeded in 24-well plates at 37°C and 5% CO_2_ for 36 h before the experiment (2 × 10^4^ cells/well). *A. baumannii* strains were grown overnight in LB at 37°C under shaking conditions. Overnight cultures were subcultured at an OD_600_ of 0.005 in LB and grown to early-stationary phase for 6 h. An appropriate volume was added to each well to reach an MOI of 100. The plates were centrifuged for 10 min at 700 × *g* to enhance bacterial contact with host cells, then incubated for 1 h at 37°C and 5% CO_2_. After 1 h of incubation, cells were washed twice with Dulbecco’s phosphate-buffered saline (DPBS) and subsequently incubated with medium supplemented with 100 µg/mL gentamicin to kill extracellular bacteria for 1 h. Then, the concentration of gentamicin was decreased to 10 µg/mL (0 h). After 4 h, cells were washed twice with DPBS and lysed with 500 µL DPBS containing 0.2% Triton X-100. Lysates were serially diluted and plated on LB agar plates to determine CFU/mL. Relative bacterial survival was calculated by dividing the number of CFU/mL at 4 h by the number of CFU/mL at 0 h.

### Intraperitoneal infection of *A. baumannii* strains in mice

For intraperitoneal inoculation in mice, freshly cultured inoculum was prepared as previously described (83). Mice were inoculated with 5 × 10^8^ CFU/mL of *A. baumannii* in 200 µL PBS using 1.0 mL BD Ultra-Fine Insulin Syringes with a 31 G needle. Groups of six infected mice were monitored for their survival post-inoculation.

### Quantification and statistical analysis

Data and statistical analyses were performed using GraphPad Prism 10.4.1. Replicates and statistical details can also be found in Materials and Methods and figure legends.

## SUPPLEMENTAL MATERIAL

Supplemental material mbio.01871-26-s0001.pdf Supplemental figures and tables.

ASM does not own the copyrights to Supplemental Material that may be linked to, or accessed through, an article. The authors have granted ASM a non-exclusive, world-wide license to publish the Supplemental Material files. Please contact the corresponding author directly for reuse.
