# Supplementary material for: IscR-mediated morphological regulation confers virulence and stress resistance by reducing stress molecule uptake in Acinetobacter baumannii
Source: mBio. 2026 Oct 6;17(11):e01871-26. doi: 10.1128/mbio.01871-26 (PMC13753788; doi:10.1128/mbio.01871-26)
Supplement: Supplementary file 1 [file mbio.01871-26-s0001.pdf]

Appendix

Supporting Information for

IscR-mediated morphological regulation confers virulence and stress resistance by reducing stress molecule uptake in *Acinetobacter baumannii*

Hoan Van Ngo[1,2,¶], Nayoung Kim[1,2,3,¶], Jumi Pak[1,2,3], Jinki Yeom[1,2,3,*]

[1] Department of Biomedical Science, College of Medicine, Seoul National University, Seoul, Korea

[2] Department of Microbiology and Immunology, College of Medicine, Seoul National University, Seoul, Korea

[3] Cancer Research Institute, Seoul National University, Seoul, Republic of Korea

* Corresponding author

E-mail: jinki.yeom@snu.ac.kr

¶These authors contributed equally to this work.

This PDF file includes:

Figures S1 to S8

Table S1 to S2

Supplementary Figures

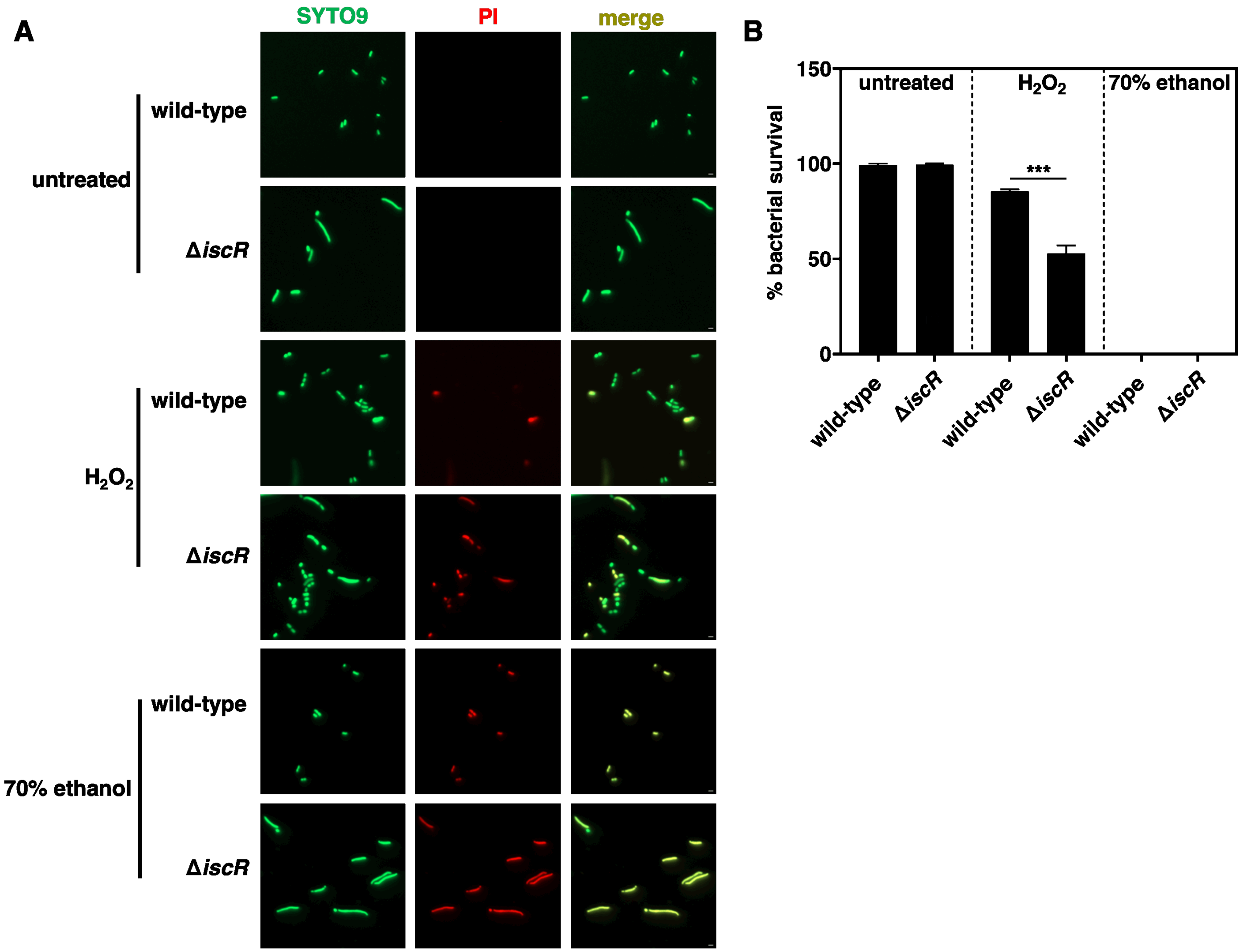

Figure S1. IscR is critical for bacterial survival under oxidative stress. (A) Fluorescence microscopy (LIVE/DEAD staining) of *A. baumannii* 17978 wild-type and the *iscR* mutant (*ΔiscR*) untreated or treated with 10 mM H_2O_2 or 70% ethanol for 2 h. Representative images from three independent experiments are shown. Both vital and dead bacteria appear green (SYTO9) and only dead bacteria appear red (PI). Scale bar = 2 µm. (B) The percentage of bacterial survival ($n \geq 500$) was calculated using ImageJ software. P values were determined using unpaired Student's t-test. ***$P < 0.001$.

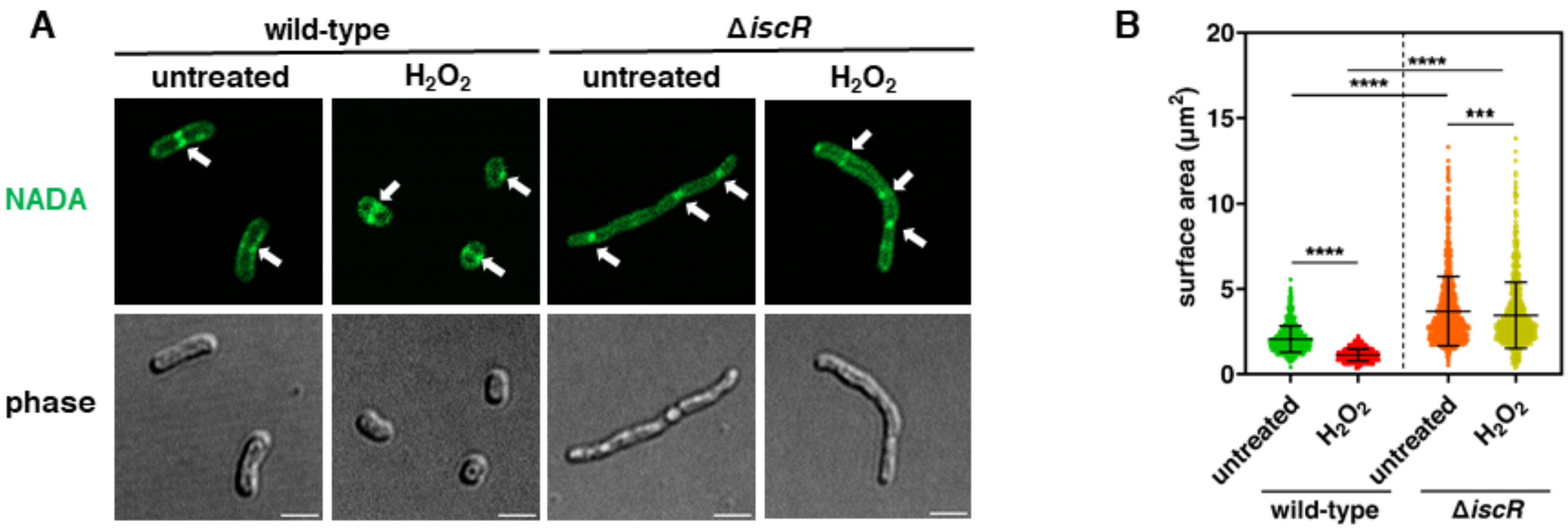

Figure S2. IscR is required for bacterial transition from rod-shaped to coccoid-shaped under oxidative stress. (A) Fluorescence (upper) and phase (lower) microscopy of *A. baumannii* 17978 wild-type and the *iscR* mutant (*ΔiscR*) in normal conditions (untreated) or treated with 10 mM H_2O_2 for 30 min. Arrows indicate bacterial septa. Scale bar = 2 μm. (B) Surface area quantifications of each cell population (n ≥ 500) were calculated using ImageJ software with the MicrobeJ plugin. Each dot represents one cell. P values were determined using two-way ANOVA with Tukey's multiple comparison test. ***P < 0.001, ****P < 0.0001.

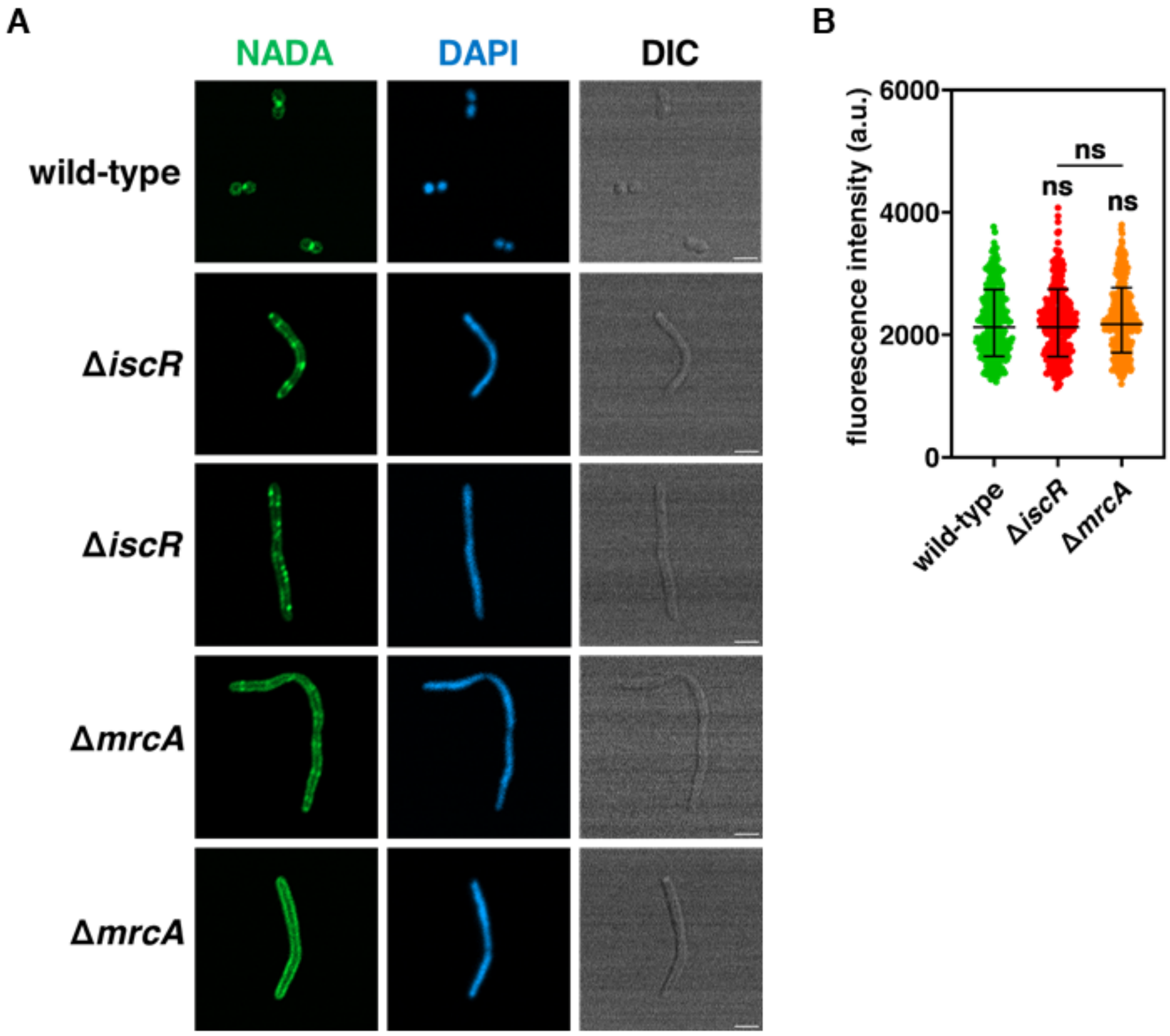

Figure S3. Inactivation of *iscR* and *mrcA* results in a single genome with multiple septa, rather than multiple genomes. (A) Confocal microscopy images of *A. baumannii* 17978 wild-type, the *iscR* (*ΔiscR*) and *mrcA* (*ΔmrcA*) mutants in normal conditions. NADA green fluorescence to detect peptidoglycan (left), DAPI for DNA detection (middle), and phase contrast for bacteria detection (right). Scale bar = 2 µm. (B) DAPI intensity of each cell population (n ≥ 300) was measured using ImageJ software with the MicrobeJ plugin. Each dot represents one cell. P values were determined using one-way ANOVA with Tukey's multiple comparison test. ns; not significant.

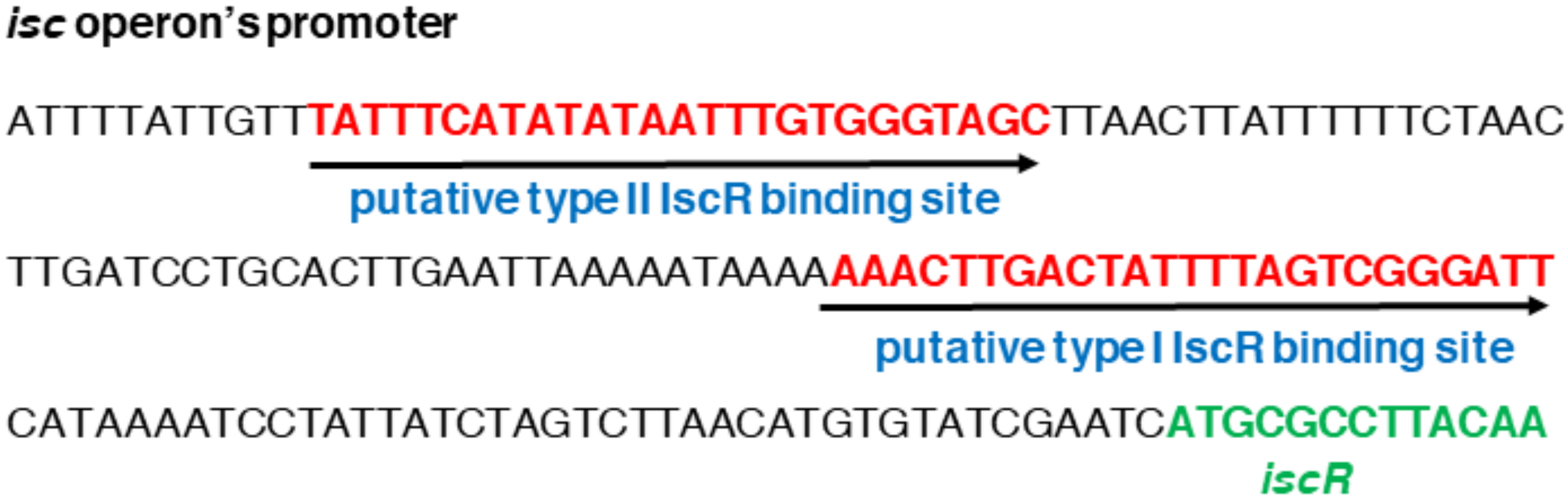

Figure S4. *A. baumannii isc* operon has putative IscR binding motifs. Putative type I and II IscR binding sites on the promoter region of the *isc* operon in *A. baumannii*.

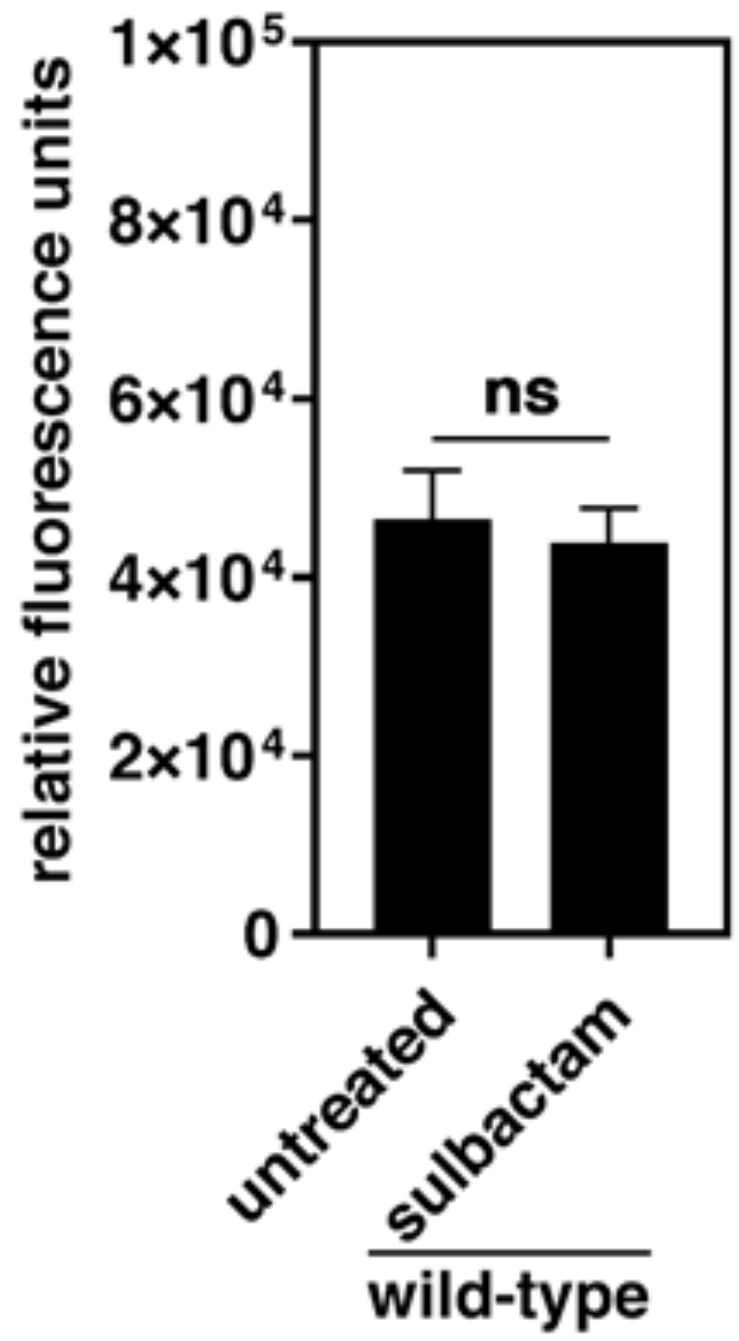

Figure S5. The sub-lethal dose of sulbactam does not significantly induce ROS inside bacteria. ROS uptake of *A. baumannii* 17978 wild-type untreated or treated with a sub-lethal dose of sulbactam (0.5X MIC) for 2 h. Data are presented as means ± SD from n = 3. *P* values were determined using unpaired Student's t-test. ns; not significant.

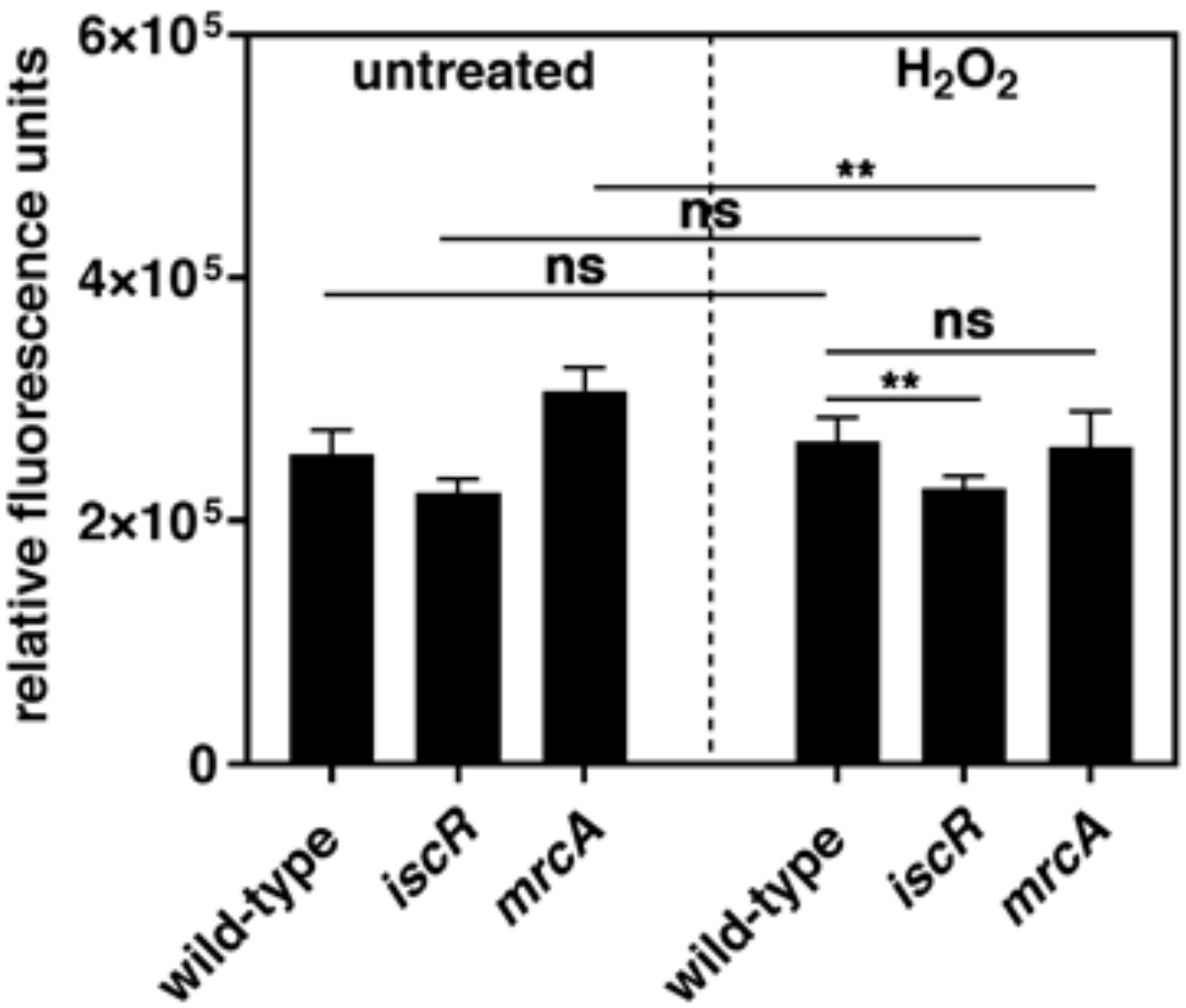

Figure S6. Membrane permeability is not affected in the *iscR* and *mrcA* mutants upon H_2O_2 treatment. Fluorescence intensity of NPN uptake in wild-type, the *iscR* (*ΔiscR*) and *mrcA* (*ΔmrcA*) mutants untreated or treated with 10 mM H_2O_2 for 2 h. Data are presented as means ± SD from n = 3. P values were determined using two-way ANOVA with Tukey's multiple comparison test. **P < 0.01, ns; not significant.

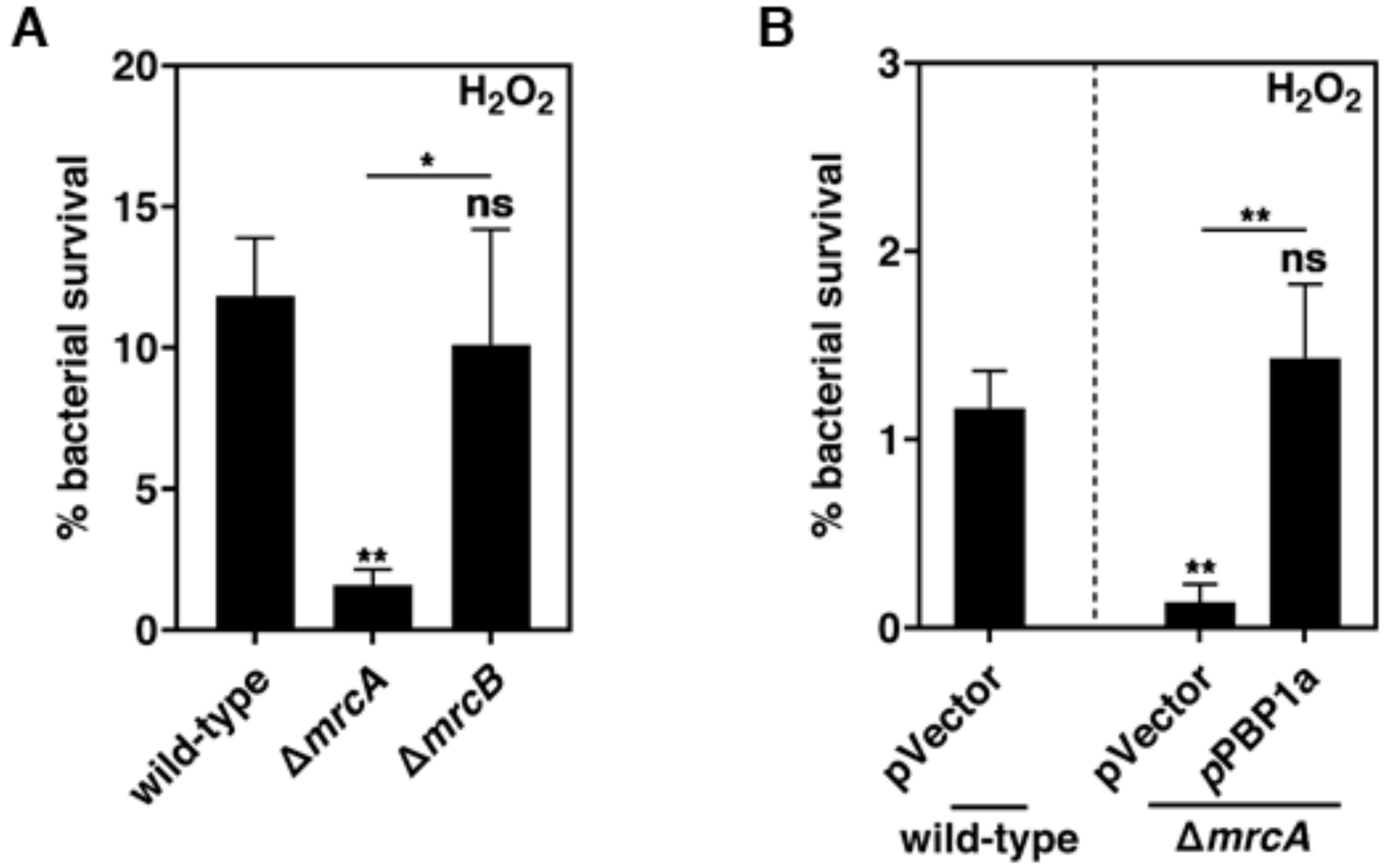

Figure S7. PBP1a, but not PBP1b, is essential for bacterial survival under oxidative stress. (A) % survival of *A. baumannii* 17978 wild-type, the *mrcA* (*ΔmrcA*) and *mrcB* (*ΔmrcB*) mutants after treatment with 10 mM H_2O_2 for 2 h. (B) % survival of *A. baumannii* 17978 wild-type harboring the plasmid vector (pVector) and the *mrcA* (*ΔmrcA*) mutant harboring the plasmid vector (pVector) or the *mrcA*-expressing plasmid (pPBP1a) after treatment with 10 mM H_2O_2 for 2 h. Data are presented as means ± SD from n = 3. P values were compared between wild-type and other strains or different combinations (A), or wild-type (pVector) and other strains or different combinations (B) using one-way ANOVA with Tukey's multiple comparison test. *P < 0.05, ** P < 0.01, ns; not significant.

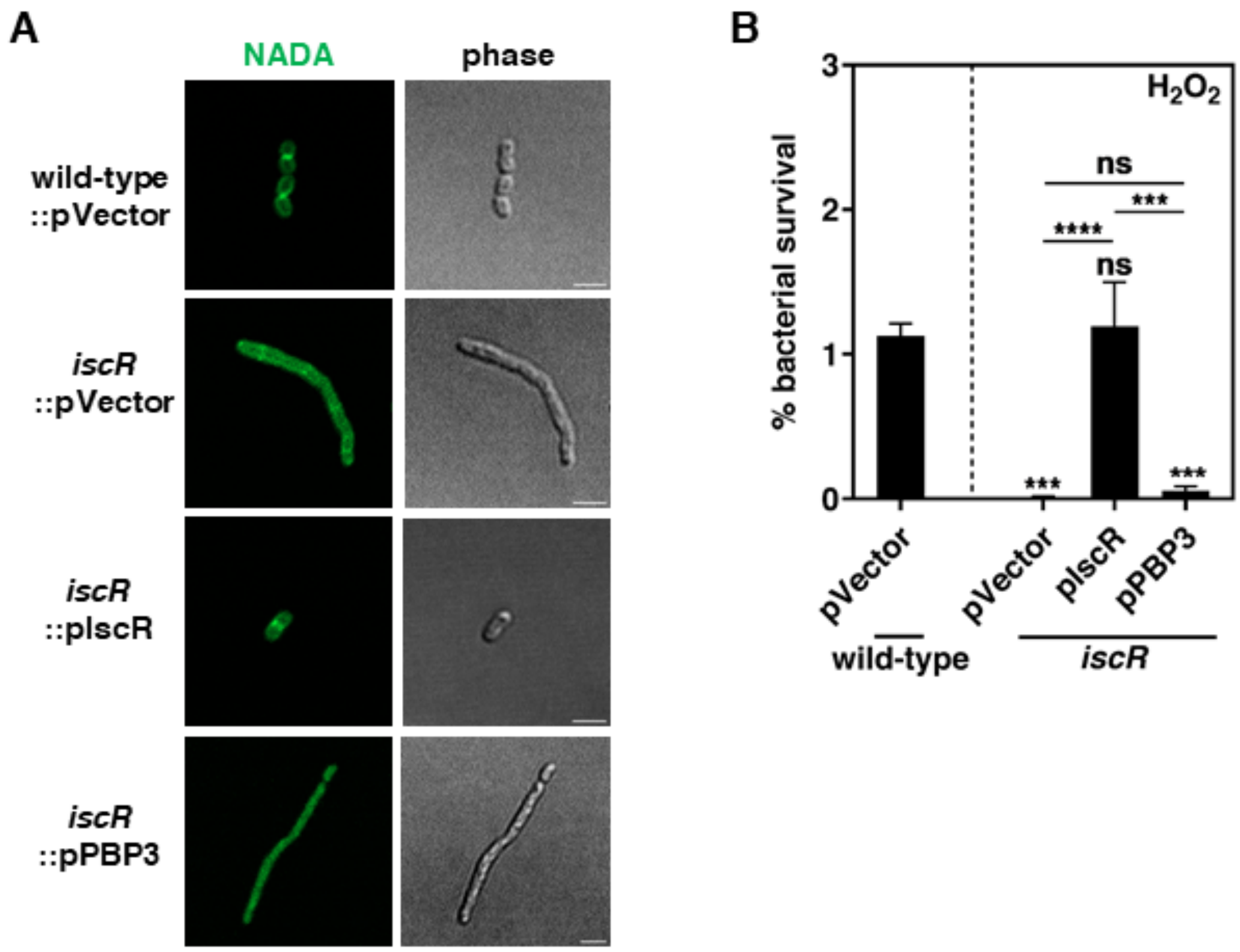

Figure S8. Complementation of the *iscR* mutant with *pbp3* could not restore defects in morphology and survival of the *iscR* mutant under oxidative stress. (A) Fluorescence (left) and phase (right) microscopy of *A. baumannii* 17978 wild-type harboring the plasmid vector and the *iscR* mutant (*ΔiscR*) harboring the plasmid vector (pVector), the *iscR*-expressing plasmid (pIscR) or the *pbb3*-expressing plasmid (pPBP3) in normal condition. Scale bar = 2 μm. (B) % survival of *A. baumannii* 17978 wild-type harboring the plasmid vector (pVector) and *iscR* mutant (*ΔiscR*) harboring the plasmid vector (pVector), the *iscR*-expressing plasmid (pIscR) or the *pbb3*-expressing plasmid (pPBP3) after treatment with 10 mM H_2O_2 for 2h. Data are presented as means ± SD from n = 3. P values were compared between wild-type (pVector) and other strains or different combinations using one-way ANOVA with Tukey's multiple comparison test. ***P < 0.001, ****P < 0.0001, ns; not significant.

Table S1. Bacterial strains and plasmids used in this study

**Strains**	**Relevant characteristics**	**Source**
Escherichia coli		
DH5a	Host strain used for generation and propagation of plasmid constructs	Life Technologies
BL21 (DE3)	Host strain used for protein expression	Novagen
Acinetobacter baumannii		
ATCC 17978	wild-type	(1)
HN310	*iscR*	This study
HN324	*mrcA*	This study
HN325	*mrcB*	This study
HN134	wild-type/pVector	This study
HN546	wild-type/pPBP1a	This study
HN314	*iscR*/pVector	This study
HN312	*iscR*/pIscR	This study
HN326	*iscR*/pPBP1a	This study
HN545	*iscR*/pPBP3	This study

HN543	*mrcA*/pVector	This study
HN544	*mrcA*/pPBP1a	This study
HN547	*mrcA*/pIscR	This study
HN548	wild-type with 3XFLAG-PBP1a	This study
HN549	*iscR* with 3XFLAG-PBP1a	This study
HN316	wild-type/pLPV1Z-P_{mrcA}	This study
HN318	*iscR*/pLPV1Z-P_{mrcA}	This study
HN489	wild-type/pLPV1Z-$P_{isc\ operon}$	This study
HN491	*iscR*/pLPV1Z-$P_{isc\ operon}$	This study
**Plasmids**		
pKD4	Template for amplification of the FRT-flanked kanamycin resistance determinant, Kan^R Amp^R	(2)
pAT02	Expresses the RecAb recombinase, Amp^R	(3)
pAT03	Expresses an IPTG-inducible flipase to remove kanamycin resistance determinant, Amp^R	(3)
pWH1266 (pVector)	*A. baumannii*/ *E. coli* shuttle and complementation vector, Amp^R	(4)

pLPV1Z	*luxCDABE* reporter plasmid, Gm^R Zeo^R	(5)
pET28b+	IPTG-inducible expression vector, Kan^R	Novagen
pET28b-IscR	pET28a+ containing full-length IscR	This study
pWH1266-*iscR* (pIscR)	pWH1266 expressing *iscR* from its native promoter, Amp^R	This study
pWH1266-*mrcA* (pPBP1a)	pWH1266 expressing *mrcA* from its native promoter, Amp^R	This study
pWH1266-*pbp3* (pPBP3)	pWH1266 expressing *pbp3* from its native promoter, Amp^R	This study
pLPV1Z-P_{mrcA}	pLPV1Z backbone, *luxCDABE* fused to *mrcA* promoter, Gm^R Zeo^R	This study
pLPV1Z-$P_{isc\ operon}$	pLPV1Z backbone, *luxCDABE* fused to *isc* operon promoter, Gm^R Zeo^R	This study

Table S2. Primers used for strain, plasmid constructions, EMSA or qRT-PCR

No.	Name	Purpose	Sequence (from 5' to 3')
1	IscR_del_F	*iscR* inactivation	GTCGGGATTCATAAAATCCTATTATCTAGTCTTAACATGTGTATCGAATCATGGTGTAGGCTGGAGCTGCTTC
2	IscR_del_R	*iscR* inactivation	GGATCTACTGGAGTGGTTGCTGCGTAATCAAGATAAATTGGACGTTTCATATCATATGAATATCCTCCTTAGT
3	PBP1a_del_F	*mrcA* inactivation	ATACTGACCACAATTAGTTTGTATTCGATCTTATTAAAGCTTACTTATCATGGTGTAGGCTGGAGCTGCTTC
4	PBP1a_del_R	*mrcA* inactivation	CTTTTTCAAAAGCGTAATAAAAAAGCCATCTAACGATGGCTTTTTTATTTTTATATGAATATCCTCCTTAGT
5	PBP1b_del_F	*mrcB* inactivation	GACAAGCTATCGCATCATTAACGCATTGTTTAGGGTTTTATCTCATTTATATGGTGTAGGCTGGAGCTGCTTC
6	PBP1b_del_R	*mrcB* inactivation	GCACCAATATAAAAAACACCTTTCTTTAACATATCCTGT

			TTCCTGTTAACGCTTATATG AATATCCTCCTTAGT
7	IscR_comp_F	cloning of *iscR* and its native promoter into pWH1266	CATGGCGACCACACCCGTC CTGTCTTATTTTTCTAACTTG ATCC
8	IscR_comp_R	cloning of *iscR* and its native promoter into pWH1266	GTTGAAGGCTCTCAAGGGC ATCGTCAAATACCTGTACC CGATAAAAG
9	PBP1a_comp_F	cloning of *mrcA* and its native promoter into pWH1266	CATGGCGACCACACCCGTC CTGTAGTCGAACTAATATCG ACACCC
10	PBP1a_comp_R	cloning of *mrcA* and its native promoter into pWH1266	GTTGAAGGCTCTCAAGGGC ATCGTTATTGCTATGACTCC CTACGC
11	PBP3_comp_up_F	cloning of *pbp3* and its native promoter into pWH1266	CATGGCGACCACACCCGTC CTGTCCCATTTTGCCAATGGA GCCATC
12	PBP3_comp_up_R	cloning of *pbp3* and its native promoter into pWH1266	GCTTTGTTCGCTTATCTACCAT CAACGGTCTCAAAAAGTAAG AC
13	PBP3_comp_down_F	cloning of *pbp3* and its native promoter into pWH1266	GTCTTACTTTTTGAGACCGTT GATGGTAGATAAGCGAACAA AGC
14	PBP3_comp_down_R	cloning of *pbp3* and its native promoter into pWH1266	GTTGAAGGCTCTCAAGGGC ATCGTTCAGGCTGGCTATAA CTTGTC

15	lux_mrcA_F	cloning promoter region of *mrcA* into pLPV1Z	CGATAAGCTTGATATCGAATTCGTCGAACTAATATCGACACCC
16	lux_mrcA_R	cloning promoter region of *mrcA* into pLPV1Z	GCTCCACCGCGGTGGCGGCCGCGAATCCCATCGGAAGTGAGAC
17	lux_isc_F	cloning promoter region of *isc* operon into pLPV1Z	CGATAAGCTTGATATCGAATTCCAACGCACCAGATGATGAAGCC
18	lux_isc_R	cloning promoter region of *isc* operon into pLPV1Z	GCTCCACCGCGGTGGCGGCCGCTGCAAAGCTAAATCAAGTAGAG
19	mrcA_EMSA_biotin_F	amplifying promoter region of *mrcA* for EMSA	Biotin-CCGTTCTTGACAGAGAGCTC
20	mrcA _EMSA_F	amplifying promoter region of *mrcA* for EMSA	CCGTTCTTGACAGAGAGCTC
21	mrcA _EMSA_R	amplifying promoter region of *mrcA* for EMSA	CATTTCCGGAAGCGTTGGGG
22	isc_EMSA_biotin_F	amplifying promoter region of *isc* operon for EMSA	Biotin-CCGAATTGCACGTTTACAACAG
23	isc_EMSA_F	amplifying promoter region of *isc* operon for	CCGAATTGCACGTTTACAACAG

		EMSA	
24	isc_EMSA_R	amplifying promoter region of *isc* operon for EMSA	GGCTGCAAAGCTAAATCAAG TAG
25	hscB_EMSA_biotin_F	amplifying promoter region of *hscB-hscA-fdx* operon for EMSA	Biotin- GACGGCTTAGAAATGGACTA TG
26	hscB_EMSA_F	amplifying promoter region of *hscB-hscA-fdx* operon for EMSA	GACGGCTTAGAAATGGACTA TG
27	hscB_EMSA_R	amplifying promoter region of *hscB-hscA-fdx* operon for EMSA	GATCTTTATCTGCGGCTTTAT C
28	IscR_pET_F	cloning of *iscR* into pET28b (+)	CATATGGCTAGCCGCCTTACA ACTCGCGGTCG
29	IscR_pET_R	cloning of *iscR* into pET28b (+)	GGCCGCAAGCTTTCAAATAC CTGTACCCGATA
30	PBP1a_3XFLAG_F	Insertion 3XFLAG tag at C-terminus of *mrcA* gene	GTCTGGTGGTCTATACTGGTG TTCTTACAGTTTGTAGACTAC AAGGACCACGACGGTGACTA CAAG GAC CAC GAC ATC GAC TACAAGGACGACGACGACAA GTGATAAGCTGGAGCTGCTTC GAAGTT

31	PBP1a_3XFLAG_F	Insertion 3XFLAG tag at C-terminus of *mrcA* gene	GTCCATAAACTACTGTAGCG GGAAAGCTTTTGTTCACGTAC CTGTTCGGGGACGGGGTAGG GTTCATACATAAATTTTGGAT GAATATCCTCCTTAGTTC

REFERENCES

1. Piechaud M, Second L. 1951. [Studies of 26 strains of Moraxella Iwoffi]. Ann Inst Pasteur (Paris) 80:97-9.
2. Datsenko KA, Wanner BL. 2000. One-step inactivation of chromosomal genes in Escherichia coli K-12 using PCR products. Proc Natl Acad Sci U S A 97:6640-5.
3. Tucker AT, Nowicki EM, Boll JM, Knauf GA, Burdis NC, Trent MS, Davies BW. 2014. Defining gene-phenotype relationships in Acinetobacter baumannii through one-step chromosomal gene inactivation. mBio 5:e01313-14.
4. Hunger M, Schmucker R, Kishan V, Hillen W. 1990. Analysis and nucleotide sequence of an origin of DNA replication in Acinetobacter calcoaceticus and its use for Escherichia coli shuttle plasmids. Gene 87:45-51.
5. Lucidi M, Visaggio D, Prencipe E, Imperi F, Rampioni G, Cincotti G, Leoni L, Visca P. 2019. New Shuttle Vectors for Real-Time Gene Expression Analysis in Multidrug-Resistant Acinetobacter Species: In Vitro and In Vivo Responses to Environmental Stressors. Appl Environ Microbiol 85:e01334-19.
